# A Multivalent mRNA Therapeutic Vaccine Exhibits Breakthroughs in Immune Tolerance and Virological Suppression of HBV by Stably Presenting the Pre-S Antigen on the Cell Membrane

**DOI:** 10.3390/pharmaceutics17020211

**Published:** 2025-02-07

**Authors:** Shang Liu, Jie Wang, Yunxuan Li, Muhan Wang, Pei Du, Zhijie Zhang, Wenguo Li, Rongchen Sun, Mingtao Fan, Meijia Yang, Hongping Yin

**Affiliations:** 1 School of Life Science and Technology, China Pharmaceutical University, Nanjing 211198, China; 1520220083@cpu.edu.cn (S.L.); 3023034219@stu.cpu.edu.cn (J.W.); 3222030959@stu.cpu.edu.cn (Y.L.); 3121030159@stu.cpu.edu.cn (M.W.); 1520220090@cpu.edu.cn (P.D.); 1520230128@cpu.edu.cn (Z.Z.); 3122034170@stu.cpu.edu.cn (R.S.); 2020221122@stu.cpu.edu.cn (M.F.); 2 Jiangsu Cell Tech Medical Research Institute Co., Ltd., Nanjing 211100, China; liwenguo@altyjy.com

**Keywords:** chronic hepatitis B, immune tolerance, hepatitis B surface antigen, mRNA therapeutic vaccines, pre-S antigen

## Abstract

**Background/Objectives**: In chronic hepatitis B infection (CHB), the hepatitis B surface antigen (HBsAg) continuously exhausts the hepatitis B surface antibody (HBsAb), which leads to the formation of immune tolerance. Accordingly, the hepatitis B virus (HBV) infection can be blocked by inhibiting the binding of the hepatitis B surface pre-S1/pre-S2 antigen to the hepatocyte receptor NTCP, but the clinical cure rate of pre-S-based vaccines for CHB is limited. **Methods**: In this study, we designed and prepared multivalent hepatitis B therapeutic mRNA vaccines encoding three hepatitis B surface antigen proteins (L, M, and S) at the cell membrane, verified via in vitro transfection and expression experiments. An in vivo immunization experiment in HBV transgenic (Tg) mice was first completed. Subsequently, an adeno-associated virus plasmid vector carrying the HBV1.2-fold genome (pAAV HBV1.2) model and the adeno-associated virus vector carrying HBV1.3-fold genome (rAAV HBV1.3) model were constructed and immunized with mRNA vaccines. The HBV antigen, antibodies, and HBV DNA in serum were detected. Indirect (enzyme-linked immunosorbent assay) ELISA were made to analyze the activated antigen-specific IgG in HBV Tg mice. Antigen-dependent T-cell activation experiments were carried out, as well as the acute toxicity tests in mice. **Results**: The L protein/pre-S antigens could be stably presented at the cell membrane with the support of the S protein (and M protein). After vaccinations, the vaccines effectively reactivated the production of high levels of HBsAb, disrupted immune tolerance, and activated the production of high-affinity antibodies against structural pre-S antigen in HBV Tg mice. The HBsAg seroconversion and serum HBV DNA clearance were achieved in two HBV mice models. Furthermore, pre-S antigen-dependent T-cell response against HBV infection was confirmed. The therapeutic vaccine also showed safety in mice. **Conclusions**: A novel therapeutic mRNA vaccine was developed to break through HBsAg-mediated immune tolerance and treat CHB by stably presenting the pre-S antigen at the membrane, and the vaccine has great potential for the functional cure of CHB.

## 1. Introduction

Hepatitis B (HBV) infection is a serious public health problem. In 2022, approximately 250 million people worldwide were infected with HBV, with a global prevalence rate of 3.2% and a prevalence rate of 5.6% in China, with more than 20 million people living with chronic hepatitis B infection (CHB). At present, only 3% to 5% of patients with CHB can be cured with interferon (IFN)-α or/and nucleoside (acid) analogs, with the majority requiring long-term medication [1]. CHB is one of the most common causes of cirrhosis and hepatocellular carcinoma [2]. Consequently, there is an urgent need for the development of functional therapeutic agents that can effectively control or cure CHB. However, the development of immune tolerance in the majority of patients and the difficulty of removing HBV covalently closed circular DNA (cccDNA) from hepatocytes have been major obstacles for the treatment of CHB.

The functional cure for hepatitis B requires the clearance of HBsAg and HBV DNA from the serum, which is the ideal treatment goal recommended by current national and international guidelines [3]. The primary challenge associated with a functional cure is finding how to disrupt immune tolerance in CHB patients. If the infection cannot be controlled by the immune system in time, it leads to the recurrent HBV infection of the hepatocytes and HBV antigens constantly stimulating the immune system, resulting in persistent chronic inflammation and immune tolerance, characterized by serum HBsAg and HBV DNA positivity, undetectable serum HBsAb, and immunosuppression of B and T-cell responses [4,5,6]. For T-cell immune tolerance, therapeutic vaccines or engineered T cells can evenly eliminate infected hepatocytes [7]. Further improvements are necessary to increase their effectiveness and safety. The presence of a considerable number of virus-like particles (VLPs) containing HBsAg in the blood is indicative of humoral immune tolerance, resulting in the exhaustion of HBsAb and B cells [6,8]. Therefore, it is extremely important to develop effective strategies for blocking the infection of HBV to reduce the pressure of numerous HBsAg on the humoral response.

Therapeutic drugs for CHB include direct antiviral drugs, which employ nucleosides or antisense oligonucleotides to impede HBV replication or transcription, and indirect antiviral drugs, which utilize immunomodulatory factors, such as interferon or PD-L1 antibodies, to stimulate the antiviral immune response. The hepatitis B therapeutic vaccine is an indirect antiviral drug. In recent years, peptide vaccines, VLPs or nanoparticles have been developed and have demonstrated favorable serologic or virologic responses in CHB therapy [9]. Among them, preS-based therapeutic vaccines have been well studied [10,11]. The domain of pre-S, containing pre-S1 and pre-S2 regions, is located in the N-terminus of the HBV surface antigen large protein (L protein). Pre-S1 was verified to mediate the recognition and binding of HBV to the hepatocyte surface receptor sodium taurocholate cotransporter polypeptide (NTCP) [12,13], and the level of pre-S1 antibodies has been reported to be significantly related to lower cccDNA levels and improved efficacy of interferon therapy in CHB patients [11,14,15]. Recently, neutralizing epitopes in the pre-S2 domain were identified, and an antibody targeting the N-terminal region of pre-S2 was shown to neutralize HBV infectivity [16]. Therefore, blocking the pre-S1 and pre-S2 domains is a promising strategy for designing efficient drugs against HBV infection [17]. However, likely due to the lack of structural antigen epitopes, pre-S-based peptides exhibit poor therapeutic efficacy in CHB patients.

Hepatitis B surface antigens are composed of L proteins, middle proteins (M protein), and small proteins (S protein or HBsAg), which are encoded by the same variable reading frame. The M protein contains a pre-S2 domain in the N-terminus and a S protein region in the C-terminus. The extension of the N-terminus of the M protein with a pre-S1 domain results in the formation of the L protein. A previous study revealed that the S protein can bind to acetyl heparin sulfate on the surface of hepatocytes and induce amidation modification of pre-S1 and that the amino acid sequence peptide at positions 2–48 in pre-S1 impedes the HBV infection of hepatocytes [12]. Consequently, the modification of pre-S, combined with the presentation of the antigen structure, is crucial for the activation of an effective and specific antibody response to HBV infection.

In vitro studies have shown that the L protein containing the pre-S1 domain is expressed and secreted weakly and causes cytotoxicity [18], whereas the pre-S2 region has no significant influence on the retention of the L protein [19]. In addition, the S protein can interact with the L protein and promote its expression and secretion [20]. According to previous reports, in addition to infectious Dane particles (complete hepatitis B virion) in the sera of CHB patients, spherical or filamentary subviral particles (SVPs) have also been found, with the latter containing the L protein, M protein, and S protein. In previous reports, serum-derived SVP was effectively used as an immunizing agent for clinical treatment [21]. Sci-B-Vac is a CHO cell-derived third-generation recombinant hepatitis B vaccine containing structural and modified L, M, and S proteins. It also can be called LMS VLP, which provides better prophylaxis and protection than HBsAg vaccines do. A previous study showed that Sci-B-Vac enhanced the efficacy of lamivudine in the treatment of CHB but failed to achieve the clinical endpoint of functional cure of CHB [22]. In addition, engineered SVPs are not stable and are prone to degradation, and the yield is generally not high [23]. Based on this, we investigated whether the structural L protein/pre-S antigen could be stably expressed and presented on the cell membrane surface to activate the immune response against HBV.

In recent years, mRNA vaccine technology has shown great potential for the prevention and treatment of diseases such as viruses and tumors, with advantages including short cycles of design and preparation, no need for additional adjuvants, and no risk of integrating exogenous nucleic acids into genomic DNA. Studies have shown that viral surface antigens can be expressed and presented on the surface of cells through delivery by mRNA vaccines, including antigen-presenting cells (APCs). In addition, antigens can be presented by MHC-I or MHC-II to CD4^+^ or CD8^+^ T cells, respectively, thereby activating B-cell or T-cell responses [24]. In addition, antigens can be directly recognized by the B-cell receptor (BCR), followed by the activation of B cells [25,26]. Consequently, we postulated that the structure of the L protein/pre-S could be expressed on the membrane through mRNA vaccine technology, activating a specific antibody response that inhibits HBV infection.

In this study, we found that upon co-expressing S (and M) proteins, the L protein/pre-S antigen can be stably expressed on the surface of VLPs or the cell membrane and that the formation of membrane raft structures can facilitate BCR binding. Furthermore, when L mRNA is at least immunized with S mRNA, mRNA vaccines can activate the production of high levels of serum HBsAb, like LMS VLP, and disrupt the tolerance of the humoral immune response mediated by high levels of HBsAg in HBV transgenic (Tg) mice. Additionally, LMS mRNA or LS mRNA effectively activated the production of high-affinity antibodies against the structural pre-S antigen. The vaccine significantly reduced the serum HBsAg and HBV DNA levels in two hepatitis B model mice, which presented high HBsAb levels. The antigen-specific antibody responses and T-cell responses were significantly activated by the vaccine. Finally, acute toxicity tests confirmed the safety of the LMS mRNA vaccine.

## 2. Materials and Methods

### 2.1. Ethics Statement and Animals

All animal care and in vivo experiments were approved by the Ethics Committee of China Pharmaceutical University (Nanjing, China). The experimental protocols for the animals conformed to the Guidelines for the Care and Use of Laboratory Animals published by the National Institutes of Health. All C57BL/6, BALB/c, or ICR mice were purchased from Jiangsu Qinglongshan Biotechnology Company (Nanjing, China). BALB/c HBV Tg mice were provided by the 458 Hospital of PLA. The animals were raised in a well-controlled room with a relative humidity of 50–60% on a 12 h light/dark cycle (6:00 h/18:00 h) and with food and water freely available.

### 2.2. Construction of Three Kinds of Hepatitis B Surface Antigen Expression Plasmids and In Vitro Transfection

The coding sequence (CDS) of the L protein (B2 isotype, DQ448619.1) was downloaded from NCBI, and sequence data could be found in Supplementary Materials. The CDS was optimized for human or murine expression preference. The transcriptional start sites of the M protein and S protein in the CDS of the L protein were mutated, as were the transcriptional start sites of the S protein in the CDS of the M protein. The CDSs of the M and S proteins were derived from the L protein. After the three genes were synthesized, each was cloned and inserted into the pD2531 plasmid containing the glutamine synthetase gene. A variety of plasmid combinations were transduced into 293T cells (8 × 10^5^ cells/well) in 6-well plates via Lipofectamine 3000 (100022234, Invitrogen Corporation, Carlsbad, CA, USA) according to the manufacturer’s protocol. The total amount of transduced plasmid in each group was 3 μg, and the mass ratio of the mixed plasmids was 1:1 or 1:1:1. The medium (A4192002, Thermo Fisher Scientific Inc., Waltham, MA, USA) containing 10% FBS (*v*/*v*) was used to culture 293T cells at 37 °C, 8% CO_2_, and 95% relative humidity (RH). The 293T cells were analyzed by flow cytometry (FCM) via Hep B preS2 (S26) antibody (sc-23944, Santa Cruz Biotechnology Inc., Dallas, TX, USA) and HBsAg antibody (20-HR20, Fitzgerald Industries International, Concord, MA, USA).

### 2.3. Expression and Purification of LMS VLP

Monoclonal LMS-A4 293F cells expressing LMS VLP were recovered via FreeStyle 293 Expression Medium (12338026, Gibco, Waltham, MA, USA), cultured in a cell culture shaker (ZWYC-290A, Nanjing ZC Bio-Tech Co., Ltd., Nanjing, China) at 37 °C, 8% CO_2_, 80% RH, and 120 rpm/min, and then progressively amplified and fermented in 2.5 L shaker flasks. The cells were precipitated via centrifugation at 600× *g* for 15 min, after which the cell precipitate was removed via centrifugation at 8000 rpm/min. The VLPs in the concentrate were purified via the Unique AutoPure Protein Purification System (Inscinstech Co., Ltd., Nanjing, China) and size exclusion chromatography (SEC) columns (HiPrep 16/60 and 26/60 Sephacryl S-400 High Resolution, Cytiva, Hogeweg, The Netherlands). Further purification was performed via a viral/biomolecule resin chromatography column (HiTrap Capto Core 400, Cytiva). The purities of the L, M, and S proteins in the VLPs were analyzed via sodium dodecyl sulfate–polyacrylamide gel electrophoresis (SDS–PAGE) and a silver staining kit (C510027, Sangon Biotech (Shanghai) Co., Ltd., Shanghai, China). The specificity of the pre-S2 antigen and S antigen was detected via Western blotting (WB). Transmission electron microscopy (TEM) was used to confirm the size and morphology of the particles, which was completed by Zhenjiang Zhuanbo Testing Technology Co., Ltd. (Zhenjiang, China). An HBsAg ELISA Kit (E-EL-H6080, Elabscience Biotechnology Co., Ltd., Nanjing, China) was used to quantify the amount of HBsAg in the VLPs.

### 2.4. Construction and Selection of LS-Expressing HEK293F Cells

The pD2531.L and pD2531.S plasmids were linearized via PvuI-HFase (10128368, New England Biolabs Inc., Ipswich, MA, USA) via the following steps. The linearized plasmids were then transfected into glutamine synthetase-deficient (GS-) HEK293F cells via PEI Prime (Serochem (P) Ltd., Pantnagar, India) according to the manufacturer’s protocol, and the culture conditions were the same as those used for HEK293F cells. One week after transfection, the transfected cells were cultured in CD293 medium (11913019, Gibco) without L-glutamine when cell viability and proliferation were favorable, and the pre-S antigen was detected by FCM after two weeks. Monoclonal cells were selected and cultured via the limited dilution method, L protein (40 kDa) was detected in the supernatants and membrane via WB using Hep B preS2 (S26) antibody, and the VLPs in the supernatants were observed via TEM (Zhenjiang Zhuanbo Testing Technology Co., Ltd.). Moreover, FCM was used to detect the pre-S antigen on the surface of monoclonal cells, and finally, two monoclonal cells, LS-4C6 293F and LS-4G2 293F, were obtained.

### 2.5. Extraction and Detection of Cell Membrane Proteins

HEK293F, LS-4C6 293F, and LS-4G2 293F cells were cultured in FreeStyle 293 Expression Medium. Once the total number of cells reached 1 × 10^7^, the cells were collected, and the cell membrane proteins and cytoplasmic proteins were extracted via the Membrane and Cytosol Protein Extraction Kit (P0033, Beyotime Biotechnology Co., Ltd., Shanghai, China). The steps included hypotonic treatment, three cycles of freezing and thawing in liquid nitrogen, centrifugation to separate membrane components from the nucleus and cytoplasm, and membrane protein lysis. The whole-cell proteins were acquired by lysing with NP-40. WB was used to analyze the L and S proteins in each fraction using the HBsAg antibody, and the ratio of L and S proteins was calculated according to the gray values of specific bands via ImageJ software (Version 13.0.6). Membrane proteins were quantified via the BCA assay, HBsAg was quantified via the HBsAg ELISA Kit, and the L protein content was calculated on the basis of the ratio of L to S. The percentage of L protein among the cell membrane proteins was also calculated.

### 2.6. Observations of the Membrane Raft Structure on the Cell Membrane

HEK293F, LS-4C6 293F, and LS-4G2 293F cells were cultured at 5 × 10^6^ and subsequently subjected to centrifugation to precipitate the cells. Then, the precipitates were fixed with 2.5% glutaraldehyde and sent to Scientific Compass (Nanjing, China) for sample preparation and TEM imaging (Zhenjiang Zhuanbo Testing Technology Co., Ltd.).

### 2.7. Preparation of LNPs/mRNAs

Novoprotein Scientific Inc. (Suzhou, China) was commissioned to perform LNP/mRNA preparation and detection. First, DNA fragments were synthesized and cloned and inserted into template plasmids according to the provided CDSs of the L protein, M protein, and S protein. The transcription templates included the T7 promoter, 5’-UTR, coding region (ORF), 3’-UTR and polyA, kanamycin resistance sequences, and so on. Then, mRNA was prepared via in vitro transcription according to protocol, which was carried out via the T7 High Yield RNA Transcription Kit with CAP1 GAG (E141, Novoprotein), resulting in a 5’-capped (Cap1-type) and 3’-tailed mRNA molecule with Pseudo-uridine triphosphate (UTP). The DNA template was removed via DNase I. mRNA was purified via lithium chloride, and the mRNA concentration and purity were detected via NanoDrop (Thermo Fisher Scientific Inc.). The length of the mRNA was detected via agarose gel electrophoresis, the cap rate and tailing were detected via LC–MS, and the integrity of the mRNA was detected via capillary electrophoresis. Liposomal nanoparticles (LNPs) were prepared according to the Moderna mRNA-1273 formulation, and the encapsulation of mRNA was performed via microfluidics. Specifically, the mRNA samples were, respectively, formulated into an aqueous phase solution at the desired final concentration using a Tris-HCl buffer (pH 7.4). Concurrently, SM-102, 1,2-distearoyl-sn-glycero-3-phosphocholine (DSPC), cholesterol, and polyethylene glycol (PEG) 2000-dimyristoyl glycerol (DMG) were mixed and configured into the total organic phase at a molar ratio of 50:10:38.5:1.5. Subsequently, parameters such as organic and aqueous phase flow rates and volumes were set on a microfluidic device for encapsulation according to the process requirements. Concentration exchange was carried out by ultracentrifugation through the pore size of the filter membrane, and a protective agent containing sucrose, acetic acid, and sodium acetate was added. Finally, the samples were filtered using a 0.22 μm filter membrane and stored at 4 °C. The particle size and polydispersity coefficient (PDI) were detected via NanoSight (Malvern Panalytical Ltd., Godalming, United Kingdom), the zeta potential (ZP) was detected via phase-analyzed light scattering, and the encapsulation rate and mRNA concentration were detected via RiboGreen (R11490, Thermo Fisher Scientific Inc.). Finally, the endotoxin content in LNPs/mRNAs was detected. The samples were stored at 4 °C and used within 6 months. Luciferase (Luc) mRNA and green fluorescent protein (GFP) mRNA were prepared via the same method and used as negative controls.

### 2.8. LNP/mRNA Transfection and Expression In Vitro

The 293T cells were spread into 6-well plates at 8 × 10^5^ cells per well overnight. Then, 5 μg of L mRNA, M mRNA, or S mRNA and 10 μg of LS mRNA (mass ratio of 1:1) or 15 μg of LMS mRNA (mass ratio of 1:1:1) were added to each well under conditions of LNP encapsulation. FCM was performed after 48 h and 96 h of transfection. A portion of the cells was removed at 48 h after transfection to extract membrane proteins, and the culture supernatants were collected at the same time. WB was performed to detect the L, M (anti-pre-S2 antigen), and S proteins (anti-S antigen) in the membrane proteins and supernatants.

### 2.9. Flow Cytometry

The cells were rinsed with precooled D- phosphate-buffered saline (D-PBS, B220KJ, BasaIMedia Technology Co., Ltd., Shanghai, China), centrifuged at 500× *g* for 5 min at 4 °C, and added to 5% normal goat serum (C-0005, Bioss Inc., Beijing, China) for 10 min on ice. After the cells were divided into two fractions, Hep B preS2 (S26) and HBsAg antibodies were added at a 1:200 dilution. After incubation for 1 h on ice, the cells were washed three times with D-PBS, and then FITC-conjugated anti-mouse (A0568, Beyotime), and FITC-conjugated anti-rabbit (A0562, Beyotime) antibodies were added. After incubation on ice for 30 min, the samples were washed three times with D-PBS and detected via a flow cytometer (NovoCyte, Agilent Technologies Inc., Santa Clara, CA, USA).

### 2.10. Western Blotting

Loading buffer (5×) was added to the cell lysates or supernatants, followed by incubation at 100 °C for 5 min and then centrifugation at 12,000 rpm/min for 5 min at 4 °C. Subsequently, 4–12% SmartPAG Precast Protein Gel Plus (SLE014, Changzhou Smart-Lifesciences Biotechnology Co., Ltd., Changzhou, China) was used for SDS–PAGE, followed by the transfer of proteins to a PVDF membrane. The PVDF membrane was blocked with 5% skim milk powder at room temperature (RT) for 1 h and then incubated overnight at 4 °C with the Hep B preS2 antibody or the HBsAg antibody diluted with tris-buffered saline with tween-20 (TBST) (1:1000). After being washed three times with TBST, the membrane was incubated with Peroxidase AffiniPure Goat Anti-Mouse IgG (33201ES60, Yeasen Biotechnology (Shanghai) Co., Ltd., Shanghai, China) or Peroxidase-Conjugated Goat Anti-Rabbit IgG (33101ES60, Yeasen) diluted in TBST (1:5000) at RT for 1 h. The membrane was then washed three times with TBST and analyzed via Super ECL Detection Reagent (36208ES76, Yeasen) in a fully automated chemiluminescence image analysis system (Shanghai Tanon Science & Technology Co., Ltd., Shanghai, China).

### 2.11. Prokaryotic Expression and Purification of Pre-S Peptides

The pre-S polypeptide was obtained via prokaryotic protein expression and purification, and the details are as follows: The *E. coli* expression-biased pre-S gene was synthesized according to the N-terminal pre-S sequence of the L protein. After the gene was cloned and inserted into the His tag in the pET:28a plasmid, the constructed plasmid was transformed into BL21 (DE3) *E. coli* (C504, Nanjing Vazyme Biotech Co., Ltd., Nanjing, China). *E. coli* was cultured in LB media supplemented with kanamycin, and the plates were coated. Colonies were selected after developing overnight, and colony PCR was conducted, followed by agarose gel electrophoresis to confirm the target bands. Positive colonies were selected via Sanger sequencing. Positive strains were recovered and amplified, and expression was induced via isopropyl-β-D-thiogalactoside (IPTG, 10902ES08, Yeasen) at a concentration of 1 mM. *E. coli* were collected and lysed, inclusion body proteins were recovered, and the target proteins were obtained via Ni column purification and SEC purification via the Unique AutoPure Protein Purification System. Endotoxin was removed from the protein mixture via Pierce High-Capacity Endotoxin Removal Resin (MAN0016351, Thermo Fisher Scientific). The purified samples were detected by nonreducing and reducing SDS–PAGE and Coomassie brilliant blue staining.

### 2.12. Immunization of HBV Tg Mice with LNPs/mRNA

HBV1.3 Tg BALB/c mice were provided by the 458 Hospital of PLA in Guangzhou, China. Fifty-five 6–8-week-old HBV1.3 Tg BALB/c mice were selected. Blood (100–200 μL) was collected from the retro-orbital venous plexus and centrifuged at 4000 rpm/min for 15 min after clotting at RT for 2 h, and the upper portion was carefully collected as the serum. Then, the serum HBsAg level was detected via an HBsAg ELISA kit. We grouped the mice equally according to serum HBsAg levels to ensure that the average HBsAg levels in each group were as close as possible, with 5 mice in each group. The groups were as follows: PBS, 10 μg Luc mRNA, 5 μg pre-S peptide + 125 μg Alhydrogel adjuvant (vac-alu-50, InvivoGen, Toulouse, France), 15 μg LMS mRNA (mass ratio of 1:1:1), 30 μg LMS mRNA (mass ratio of 1:1:1), 10 μg LS mRNA (mass ratio of 1:1), 20 μg LS mRNA (mass ratio of 1:1), 5 μg L mRNA, 10 μg L mRNA, 10 μg S mRNA, and 5 μg LMS VLP + 125 μg Alhydrogel adjuvant. The immunization and assay protocols were as follows: each drug was administered via intramuscular injection four times starting on Day 0, week 2, week 4, and week 16. Blood was collected and tested every 2 weeks during the first 20 weeks and every 4 weeks from the 20th week to the 32nd week. After serum separation, KingMed Diagnostics (Nanjing, China) was commissioned to detect serum HBsAg and HBsAb via an Chemiluminescence immunoassay (CLIA).

### 2.13. Indirect Enzyme-Linked Immunosorbent Assay

For the HBV Tg mouse immunization experiments, blood was collected, and the serum was separated at weeks 9, 11, 13, and 15. The sera of mice treated with the same kind of vaccine were mixed separately, and the IgG in the serum was purified via protein A columns in a unique AutoPure protein purification system. The purity and specificity of the IgG were determined via SDS–PAGE and WB. Subsequently, an indirect enzyme-linked immunosorbent assay (ELISA) was used to detect the binding ability of IgG to different hepatitis B antigens as follows: a. Pre-S peptide and recombinant HBsAg (671-01, Shanghai PrimeGene Bio-tech Co., Shanghai, China) were diluted to 1 μg/mL in NaHCO_3_ buffer (pH 9.6), and the LMS VLP were diluted to 10 μg/mL. These dilutions were subsequently added to the plate after 0.5 h of UV irradiation. b. The plate was washed with 300 μL/well of PBS 3 times, after which 200 μL of 5% normal goat serum was added, and the mixture was incubated at RT for 2 h. c. One microgram of IgG was added to each well (3 replicate wells per group) and incubated at 4 °C overnight. d. The plate was washed with 300 μL/well with PBST 3 times, and 100 μL of a 1:5000 dilution of peroxidase AffiniPure goat anti-mouse IgG was added and incubated for 2 h at RT on a shaker. d. The plate was washed with 300 μL/well with PBST 3 times, and 100 μL/well TMB One Solution (36602ES60, Yeasen) was added and incubated for 10 min, followed by the addition of 50 μL of 2 M H_2_ SO_4_ termination solution, which was detected by an enzyme marker (Thermo Fisher Scientific Inc.), and the OD450–OD603 values were calculated. In addition, IgG samples from the LMS mRNA, LS mRNA, LMS VLP, and pre-S groups were gradient diluted to nine concentrations, and the binding of IgG to the pre-S peptide was detected via indirect ELISA and analyzed via GraphPad Prism software (Version 9.5.1) via the variable slope (four parameters). The data were analyzed via nonlinear regression (curve fit) via GraphPad Prism software to estimate the EC50 values.

### 2.14. Immunization of pAAV-Induced HBV-1.2 Mice with LNPs/mRNA

Seventy-two 5–6-week-old male C57BL/6J mice were purchased to construct the pAAV HBV1.2 model. Eight micrograms of pAAV HBV1.2 plasmid were injected into C57BL/6J mice via the tail vein at a volume of 8% of the body weight (approximately 1.6 mL) within 8 s. Blood was collected, and the serum was separated after 6 weeks of modeling, after which the serum HBsAg levels were detected. Twenty-four mice with relatively close levels of HBsAg were selected and equally divided into 4 groups (n = 6) according to the average serum HBsAg level. Then, the mice were immunized with 10 μg of GFP mRNA, 10 μg of SmRNA, and 6 μg or 15 μg of LMS mRNA (mass ratio of 1:1:1) via intramuscular injection on Days 0 and 14. Serum HBsAg, HBsAb, and HBeAg were detected each week. The serum HBV DNA of the mice at week 0 and week 5 were detected via quantitative real-time PCR (q-PCR).

### 2.15. Immunization of rAAV8 HBV1.3 Mice with LNPs/mRNAs

A sufficient number of 5–6-week-old male C57BL/6J mice were purchased to construct the rAAV8 HBV1.3 model. Each mouse was injected with 1 × 10^10^ vg (200 μL) of rAAV8 HBV1.3 virus solution (ayw D type, Beijing FivePlus Molecular Medicine Institute Co., Ltd., Beijing, China) via the tail vein. The serum was separated after 6 weeks of modeling to detect the serum HBsAg level. Mice with relatively close HBsAg levels were selected and grouped equally (n = 7). Then, the mice were immunized with 9 μg of GFP mRNA, 10 μg of S mRNA, 3 μg or 9 μg of LMS mRNA (mass ratio of 1:1:1), and 2 μg or 6 μg of LS mRNA (mass ratio of 1:1) via intramuscular injection on Days 0, 14, 28, and 49. Serum HBsAg, HBsAb, and HBeAg were detected every 2–3 weeks. q-PCR was performed to detect serum HBV DNA at week 0 and week 9. In addition, the rAAV8 HBV1.3 model was constructed using 5 × 10^10^ vg (200 μL) of the rAAV8 HBV1.3 viral mixture and was immunized with 1 μg, 3 μg, or 9 μg of LMS mRNA (mass ratio of 1:1:1), with the 9 μg of GFP mRNA group and 1 μg of S mRNA group used as controls. The levels of antigen and antibody were detected and analyzed in the same way.

### 2.16. Quantification of Serum HBV DNA by q-PCR

The HBV DNA in the serum of mice was detected via an HBV Nucleic Acid Quantification Kit (PCR-fluorescent probe method) from Sansure Biotech Inc. (Changsha, China). The kit uses the magnetic bead to extract HBV DNA from samples and utilizes specific primers and a specific fluorescent probe (TaqMan probe, 5′-end labeled with FAM fluorescein) designed for the conserved DNA region of hepatitis B virus. Additionally, there is a positive internal reference control (inactivated hepatitis B virus standard) used with the TaqMan probe, 5′-end labeled HEX fluorescein, to avoid false negatives. The kit also includes an internal reference fluorescent ROX for more accurate quantification. According to the protocol, the main steps are as follows: first, different DNA extracts were added to serum samples or negative control, positive control, quantitative reference samples several times, and the eluted DNA solution was obtained after several mixing and centrifugation. Then, the PCR mix was added to DNA solution separately and mixed followed by centrifugation. Finally, the parameters of PCR were set in LineGene 9600 Plus fluorescence q-PCR detector (Bioer Technology, Hangzhou, China), selecting the detection fluorescence channel (FAM), the internal standard detection channel (HEX), and the reference fluorescence (ROX). The program was set to 1 cycle: 50 °C for 2 min, 95 °C for 2 min, 2 cycles: 95 °C for 15 s, 58 °C for 30 s, and 1 cycle: 25 °C for 10 s. A standard curve was made based on the results of the four HBV quantitative reference samples, and the concentration of HBV DNA was calculated with a lower limit of detection of 10 IU/mL.

### 2.17. Analysis of Antigen-Dependent T-Cell Activation

First, 6–8-week-old male BALB/c mice were immunized with 50 μL of PBS, 3 μg of LMS mRNA (mass ratio of 1:1:1), 2 μg of LS mRNA (mass ratio of 1:1), or 1 μg of S mRNA, and antigen-specific T-cell activation was analyzed on Day 14. BALB/3T3 cells cultured in DMEM containing 10% FBS (*v*/*v*) were spread on 6-well plates at 5 × 10^5^/well, followed by transient cotransfection with pD1531.L, pD2531.M, and pD2531.S plasmids (mass ratio of 1:1:1) into BALB/3T3 cells. FCM was used to detect pre-S antigen on the cell surface 48 h later. The immunized BALB/c mice were sacrificed after 14 days, and the spleens were isolated and ground. Lymphocytes were isolated via Percoll density gradient centrifugation, and then T cells were isolated via the MagniSort Mouse CD3 T-Cell Isolation Kit (480031, BioLegend, Inc., San Diego, CA, USA) according to the manufacturer’s instructions. After counting, the cells were resuspended in T-cell culture medium containing X-Vivo 15 (04-744) containing retrans medium (BEBP02-054Q, Lonza Group Ltd, Basel, Switzerland), 2 mM glutamine, 1 mM sodium pyruvate, 55 mM β-mercaptoethanol, and MEM nonessential amino acid (NEAA, 11140050, Gibco). A total of 5 × 10^5^ T cells from each group were added to the transfected BALB/3T3 cells at 5 × 10^5^/well and incubated for 24 h in a mixture of media from both cell lines (1:1). Brefeldin A (420601, BioLegend) was added at a concentration of 5 μg/mL 4 h before the end of the experiment. Inactivated and unincubated T cells were used as negative controls, and T cells activated with CD3/CD28 beads (11452D, Gibco) were used as positive controls, with three replicates for each treatment. T cells were obtained by rinsing with precooled PBS and blocking with 5% (*v*/*v*) goat gamma globulin (005-000-002, Jackson ImmunoResearch Laboratories, Inc., West Grove, PA, USA) at 4 °C for 30 min. Then, CD3e monoclonal antibodies (145-2C11) and APC (17-0031-82, Invitrogen) were added, and the mixture was incubated for 1 h. After 3 washes with PBS, the cells were permeabilized with True-Phos Perm Buffer (425401, BioLegend) and incubated with IFN gamma monoclonal antibodies (XMG1.2) and FITC (11-7311-81, Invitrogen). After washing, FCM was used to detect intracellular IFN-γ expression in T cells. The APC-conjugated Armenian hamster IgG isotype control (17-4888-81, Invitrogen) and FITC-conjugated rat IgG1 kappa isotype control (11-4301-81) antibodies were used as isotype controls, respectively.

### 2.18. Acute Toxicity Test in Mice

Five- to six-week-old male ICR mice were prepared and injected intramuscularly with 1.5 μg, 7.5 μg, or 37.5 μg of LMS mRNA (mass ratio of 1:1:1) on Days 0 and 3, with an equal volume of PBS used as a control (n = 8). During the experiment, the mice were observed every 3–4 days for death, and appearance, behavioral activity, and body weight changes were recorded up to 21 days after the second administration. At the end of the experiment, the serum was separated, and the main organs of the mice, including the heart, liver, spleen, lungs, and kidneys, were weighed. After the organs were rinsed with PBS and fixed in 4% paraformaldehyde for 2 days, FreeThinking (Nanjing, China) was used to carry out H&E staining for pathological analysis. KingMed Diagnostics was used to detect liver and renal function in the serum of the mice. Liver function indices included alanine aminotransferase (ALT) and azelaic aminotransferase (AST), and renal function indices included urea, uric acid (UA), and creatinine (CR).

### 2.19. Statistical Analysis

The EC50 values were determined by fitting to a variable slope (four parameters) via nonlinear regression (curve fit) analysis via GraphPad Prism. The differences among multiple groups were analyzed by one-way analysis of variance (ANOVA) with Dunnett’s test or, if appropriate, repeated-measures ANOVA with Bonferroni’s post hoc correction. The differences between two groups were compared via the unpaired Student’s *t*-test. Values of *p* < 0.05 and *p* < 0.01 were considered significant, and ns represented not significant.

## 3. Results

### 3.1. L Protein/Pre-S Could Be Stably Expressed in VLPs and the Cell Membrane with the Support of the S Protein and M Protein

As showed in Figure S1, the pre-S region of the L protein is indispensable for the activation of antibody responses against HBV infection in hepatocytes [27], and it has been determined that LMS VLP constitute a proportion of the L protein [23]. Consequently, we initially examined the quantity of L protein present in the LMS VLP, which was obtained through fermentation and purification. WB and SDS–PAGE analyses revealed that the content of L protein in the LMS VLP was approximately 10% of the S protein content (Figure S2a–c). TEM revealed that the size of the LMS VLP was in the range of 20–30 nm, with a regular round shape (Figure S2d). Furthermore, we sought to determine whether VLPs could be secreted without M proteins. The pD2531.L and pD2531.S plasmids were co-transfected into GS- HEK293F cells. Gln-deficient conditions were used to select the cells for monoclonality. L protein was detected in the supernatants of seven monoclonal cells (Figure S3a). TEM analysis revealed that these monoclonal cells secreted different amounts and sizes of VLPs, with sizes ranging from 15 to 45 nm (Figure S3b). LS-9G8 293F monoclonal cells, which exhibited optimal cellular status and homogeneous VLP particles, were selected for fermentation. Despite successful secretion, acquiring high-purity VLP was challenged.

During the expression and secretion of LMS VLP, hepatitis B surface antigen proteins undergo membrane transport through the endoplasmic reticulum and Golgi apparatus [20]. However, it is unclear whether these three proteins can be expressed and presented on the cell membrane. Accordingly, we transfected the expression plasmids of the three proteins (Figure S4a) transiently into 293T cells in different combinations, and the ratio of the expression of the pre-S antigen and the S antigen on the surface of the cells was detected via FCM. The results demonstrated that the L, M, LS, MS, and LMS transfectants were all capable of expressing pre-S antigen (Figure S4b), as well as the S antigen. As observed in previous reports, cytotoxicity was markedly elevated when pD2531.L was transfected alone.

L protein is expressed and secreted weakly on its own and is somewhat cytotoxic, whereas when it is co-expressed with the S protein and M protein, L proteins affect the secretion of the latter two proteins [18,19]. The percentage of pre-S antigen-positive cells on the surface of monoclonal cells that secreted LMS VLP was approximately 45%, as determined by FCM (Figure S4c), indicating that the S protein, M protein, and L protein can be expressed on the cell membrane. It remains unclear whether it is possible to stably express L proteins on the cell membrane with the support of S proteins only. Stable transfection of pD2531.L and pD2531.S plasmids into GS- HEK293F cells, or transfected pD2531.L alone. After pressurized screening, FCM analysis of the pre-S antigen revealed that both L and LS were capable of expressing L proteins (Figure S5a). Continued culture revealed that the cell viability was poor for L transfection alone, while monoclonal cells could only be successfully selected from LS transfection. As shown in Figure S5b, the L protein was present on the cell membrane of multiple monoclonal cells, which was further verified by WB (Figure S5c). Monoclonal cells, including LS-4C6 293F and LS-4G2 293F cells, were selected for fermentation. Following multiple passages, a slight decrease in pre-S positivity was observed, with a value of approximately 50%, as determined by FCM (Figure S6a). Subsequently, distinct cell fractions were extracted and isolated, and the WB (anti-S antigen) assay demonstrated that both cell membranes contained S protein (approximately 25 kDa) and L protein (approximately 40 kDa), as illustrated in Figure S6b. Gray-scale analysis demonstrated that the L protein constituted approximately 39% and 28% of the S protein. The concentration of membrane protein was quantified via BCA, and HBsAg was quantified via ELISA and converted to the L protein content according to the ratio of the two proteins. The L protein was calculated to constitute approximately 15.7% and 1.7% of the membrane proteins.

When some viral surface antigens are expressed on cell membranes, membrane rafts can form, usually presenting a density of antigens, which can enhance the binding of the antigen to BCR [28]. TEM observations of LS-4G2 293F and LMS-A4 293F cells revealed that many more membrane rafts were found on the surface of both cell lines than on the surface of HEK293F cells (Figure S6c), indicating that the co-expression of L and S proteins, or L, M, and S proteins, can result in the formation of membrane rafts on the surface of cells.

**Figure 1:**
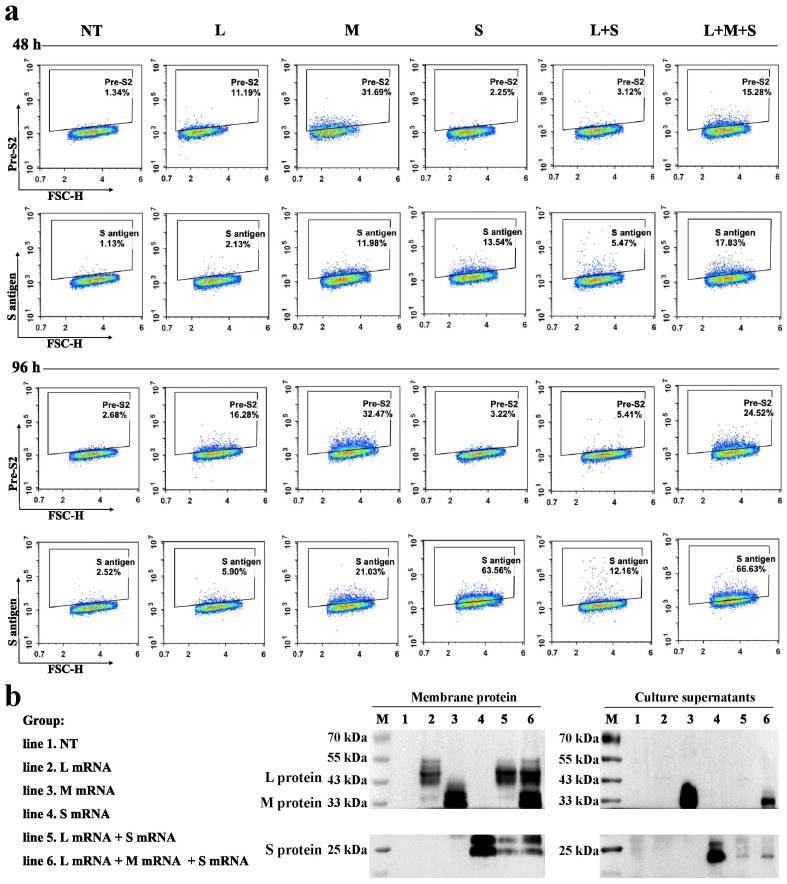
In vitro expression analysis of hepatitis B surface antigen mRNA vaccines. LNP-encapsulated L mRNA, M mRNA, and S mRNA were transfected into 293T cells in different combinations, and the dosage of each single mRNA was 5 μg for each treatment. (**a**) The proportion of cells expressing pre-S2 antigen or S antigen on the surface was detected by FCM at 48 h and 96 h after transfection, respectively. The results of FCM were showed using density maps. (**b**) WB analysis of the expression or secretion of the L protein (42 kDa), M protein (31 kDa), and S protein (27 kDa) in the cell membrane or supernatants at 48 h after transfection.

mRNA vaccines designed on the basis of viral surface antigens can be expressed and presented on cell membranes to activate adaptive immune responses in multiple ways [26]. Accordingly, we prepared mRNAs encoding three hepatitis B surface antigen proteins (L mRNA, M mRNA, and S mRNA) with LNP encapsulation, respectively, and the characterization data are shown in Table S1. The encapsulation rate of each LNP/mRNA was greater than 90%. Physicochemical characterization revealed that the average particle size of all three LNP/mRNAs was around 80 nm, and the PDI value indicated that the particle sizes were relatively homogeneous, and ZP analysis showed that the particle surfaces were weakly negatively charged (−3 mV to −8 mV). The 293T cells were transfected with different combinations of LNPs/mRNAs, and the expression of antigens on the cell membranes was detected via FCM at 48 and 96 h. As illustrated in Figure 1a, the levels of protein translated from the mRNAs in each group notably increased from 48 h to 96 h. The M mRNA-transfected group presented the highest percentage of cells expressing the pre-S antigen, followed by the three mRNAs that were co-transfected. However, the positivity rate was significantly lower when L was co-transfected with S than when L was transfected alone. Furthermore, the highest rate of S antigen positivity was observed in LMS mRNA co-transfection, with a higher percentage than that of the pre-S antigen. WB analysis, as shown in Figure 1b, revealed that the membrane expression level of the L protein was significantly greater in cells co-transfected with LMS and LS than in those transfected with L alone. The M protein was identified in the cell culture supernatant following transfection with M or LMS, and the S protein was detected in both cell lines following transfection with S mRNA. In addition, TEM observation of the supernatants did not reveal VLPs.

### 3.2. Co-Formulated mRNA Vaccines Encoding Hepatitis B Surface Antigens Breaks Through Humoral Immune Tolerance in HBV Tg Mice

**Figure 2:**
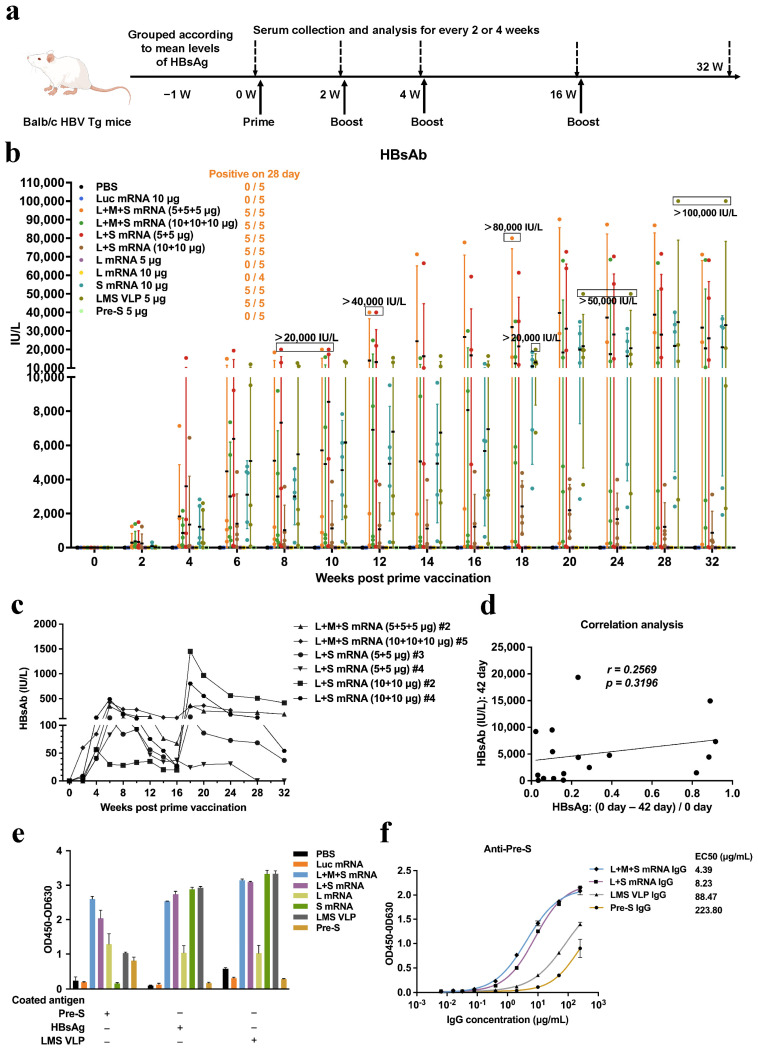
Humoral immune response analysis of HBV Tg mice immunized with the hepatitis B surface antigen mRNA vaccines. (**a**) Schematic diagram of the experimental protocol for the immunization of BALB/c HBV Tg mice with different combinations of mRNAs encoding hepatitis B surface antigens or with control vaccines. (**b**) CLIA detection of serum HBsAb levels in HBV Tg mice at weeks 0–32 of the immunization experiment. (**c**) Analysis of changes in the serum HBsAb concentration in mice whose HBsAb concentration was consistently less than 2000 IU/L and greater than 0 IU/L. (**d**) Correlation analysis between the serum levels of HBsAb and reduced HBsAg levels. (**e**) Indirect ELISA analysis of the binding ability of induced serum IgG to recombinant HBsAg, the pre-S peptide, or the LMS VLP after immunization with different vaccines. (**f**) Variable slope (four parameters) analysis via nonlinear regression (curve fit) was performed for the pre-S antigen-specific indirect ELISA. Representative results are presented as the means ± standard deviations (SDs).

In CHB patients with high serum levels of HBsAg, breaking immune tolerance with therapeutic vaccines such as HBsAg, pre-S, or LMS VLP is difficult, which is due to their inability to effectively activate antibody responses that specifically block HBV infection. Based on these results, the presentation of natural pre-S antigens on the cell membrane through mRNA technology may be a more efficacious approach. Therefore, we immunized HBV Tg mice with varying serum HBsAg levels (500 IU/mL to 50,000 IU/mL) and immune tolerance statuses, with different combinations of mRNAs encoding hepatitis B surface antigens, and we used PBS, Luc mRNA, and purified pre-S peptide (Figure S7) as controls. The experimental protocol for immunization is shown in Figure 2a and refers to the research on LMS VLP [29].

The results of the serum HBsAb analysis are presented in Figure 2b and Figure S8. Two weeks after the initial immunization, HBsAb was detected in all the mice vaccinated with a high dose of LMS mRNA and in 80% of the mice vaccinated with a low dose of LMS mRNA. Additionally, 60% to 80% of the mice immunized with LS mRNA or S mRNA exhibited production of HBsAb, and the former had higher antibody levels. Two weeks after the second vaccination, serum HBsAb was found in all the mice vaccinated with LMS mRNA, LS mRNA, or S mRNA, with antibody levels exceeding 10 IU/L, which was achieved in all the mice immunized with LMS VLP at week 6. Following the third immunization, which commenced at week 14, HBsAb levels exceeded 40,000 IU/L in mice immunized with LMS mRNA or LS mRNA. After the last immunization at week 16, high levels of HBsAb were maintained until the end of the experiment at week 32. Analysis of the antibody levels in the mice with lower serum HBsAb concentrations (consistently less than 2000 IU/L and greater than 0 IU/L) revealed that, with the exception of one mouse that cleared HBsAb, the HBsAb concentrations of the other mice remained higher than 10 IU/L (Figure 2c). Notably, HBsAb was consistently undetectable in the sera of the mice vaccinated with L mRNA, PBS, or pre-S peptide. Furthermore, our findings revealed that the serum HBsAg levels did not tend to decrease across all the mice (Figure S9). We suggest that the situation is attributed to the fact that the majority of cells in HBV Tg mice persistently express high levels of serum HBsAg. The results in Figure 2d revealed that there was no significant correlation between reduced serum HBsAg and activated HBsAb, indicating that high levels of HBsAg do not influence the production of HBsAb in vaccinated HBV Tg mice.

To verify whether the vaccines activated specific antibody response against the pre-S antigen, we purified serum IgG from each group and used indirect ELISA to analyze the binding of IgG to different antigens, including the pre-S peptide, recombinant HBsAg, and LMS VLP containing pre-S. The results are shown in Figure 2e. The LMS mRNA, LS mRNA, and LMS VLP vaccines significantly induced the production of antibodies against both the pre-S antigen and the S antigen. Additionally, immunization with L mRNA (which contains the S antigen fragment) alone resulted in limited production of anti-pre-S and anti-HBsAg antibodies. Furthermore, IgG induced by the pre-S peptide did not recognize the LMS VLP, indicating that the linearized pre-S peptide did not activate IgG response against the structural pre-S antigen in VLP. Furthermore, the titer analysis of IgG revealed that the IgG induced by LMS mRNA and LS mRNA had a more robust ability to bind to the pre-S antigen than the LMS VLP and pre-S peptide vaccines did (Figure 2f).

In summary, in HBV Tg mice with hepatitis B immune tolerance, LMS mRNA and LS mRNA can sustainably activate high levels of serum HBsAb and break HBsAg-mediated humoral immune tolerance, activating specific antibodies against structural pre-S antigens.

### 3.3. Hepatitis B Surface Antigen mRNA Vaccines Induce Seroconversion and Viral Suppression in HBV Mice

To further assess the potential of hepatitis B surface antigen mRNA therapeutic vaccines to stimulate an anti-HBV immune response, two commonly used mouse models of hepatitis B, the pAAV HBV1.2 and rAAV8 HBV1.3 models, were selected for investigation.

**Figure 3:**
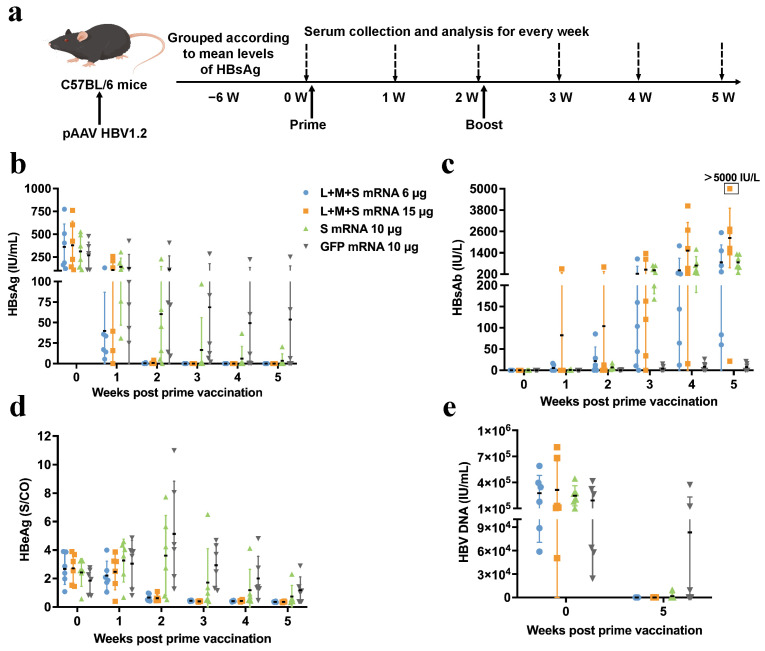
Serological and virological response analysis of pAAV HBV1.2 mice immunized with hepatitis B surface antigen mRNA vaccines. (**a**) Schematic diagram of the experimental protocol for immunization of pAAV HBV1.2 mice with LMS mRNA vaccines, S mRNA, or GFP mRNA. Changes in serum (**b**) HBsAg, (**c**) HBsAb, and (**d**) HBeAg from 0 to 5 weeks after immunization of pAAV HBV-1.2 mice with different vaccines. (**e**) Q–PCR detection of serum HBV DNA levels in immunized mice at week 0 and week 5. Representative results are presented as the means ± SDs.

First, a pAAV HBV1.2 mouse model with relatively short expression of hepatitis B antigen was constructed for immunization experiments (Figure 3a). Based on the results of the immunization of HBV Tg mice, LMS mRNA was selected for vaccination at a lower dose, and S mRNA and GFP mRNA were used as controls. The results shown in Figure 3b,d demonstrated that mice immunized with LMS mRNA were negative for serum HBsAg and HBeAg in the third week. By the fifth week, hepatitis B antigen in all but one mouse had become negative in the S mRNA-vaccinated group. The analysis of serum HBsAb levels demonstrated that LMS mRNA induced the production of high levels of HBsAb at an earlier stage than did S mRNA (Figure 3c). At the end of the experiment, serum HBV DNA was undetectable or nearly undetectable in all the mice immunized with LMS mRNA (Figure 3e).

**Figure 4:**
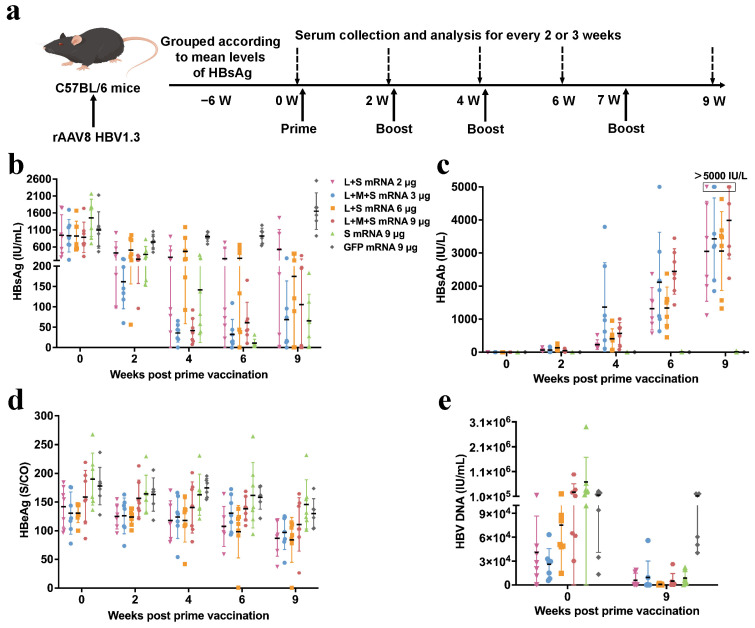
Serological and virological response analysis of rAAV8 HBV1.3 mice immunized with hepatitis B surface antigen mRNA vaccines. (**a**) Schematic diagram of the experimental protocol for immunization of rAAV8 HBV1.3 (1 × 10^10^ vg/mouse) mice with hepatitis B surface antigen mRNA vaccines in different combinations, S mRNA, or GFP mRNA. Changes in serum (**b**) HBsAg, (**c**) HBsAb, and (**d**) HBeAg from 0 to 9 weeks of immunization with different vaccines. (**e**) Q–PCR detection of serum HBV DNA levels in immunized mice at week 0 and week 9. Representative results are presented as the means ± SDs.

We subsequently constructed a rAAV8 HBV1.3 mouse model using a 1 × 10^1^⁰ vg recombinant virus and performed immunization experiments with the protocol illustrated in Figure 4a. The results of antigen detection demonstrated that the majority of the mice immunized with LMS mRNA, LS mRNA, and S mRNA, except for the GFP mRNA, presented significantly reduced serum HBsAg (Figure 4b and Figure S10). However, the most pronounced HBsAg suppression effect was observed at the sixth week. The serum HBsAb results demonstrated that either LMS mRNA or LS mRNA resulted in higher antibody levels, and the former performed better (Figure 4c). Although S mRNA did not significantly induce the production of HBsAb, it clearly affected HBsAg, indicating that it might trigger a more robust T-cell immune response. Furthermore, immunization with LMS or LS mRNA resulted in the suppression of HBeAg expression in a subset of mice (Figure 4d). Ultimately, the serum HBV DNA levels were markedly diminished in all the mice immunized with hepatitis B surface antigen mRNAs (Figure 4e).

To further determine whether the immune response is dose dependent, we constructed a rAAV8 HBV1.3 model using 5 × 10^1^⁰ vg recombinant virus and compared the immunization effects of different doses of LMS mRNA (Figure S11a). The results demonstrated that immunization with 1 μg of LMS mRNA or S mRNA did not inhibit serum HBsAg. In contrast, immunization with 3 μg or 9 μg of LMS mRNA had effects on the inhibition of HBsAg, with the former being more effective (Figure S11b,c). Further analysis revealed that 1 μg of S mRNA was unable to activate HBsAb, whereas 1 μg, 3 μg, or 9 μg of LMS mRNA significantly activated HBsAb (Figure S11d). Furthermore, a significant correlation was observed between the reduction in HBsAg and the level of HBsAb (Figure S11e). Additionally, no notable change in the serum HBeAg level was detected (Figure S11f).

The results of the immunization experiments in the two hepatitis B mouse models demonstrated that the LMS mRNA vaccine and the LS mRNA vaccine exhibited comparable efficacy in inhibiting HBsAg and HBV DNA, along with more pronounced production of HBsAb, than the S mRNA vaccine. Furthermore, the T-cell immune response may play a pivotal role in the clearance of HBsAg.

### 3.4. Hepatitis B Surface Antigen mRNA Vaccines Activate Antigen-Dependent T-Cell Responses

**Figure 5:**
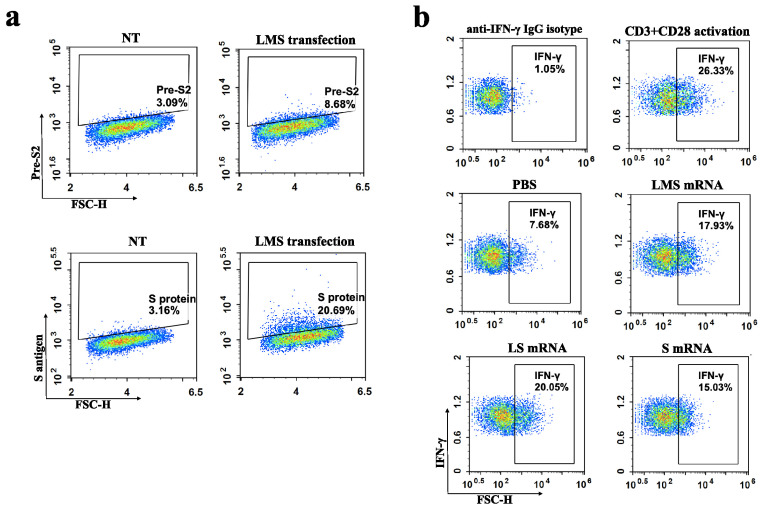
The analysis of antigen-dependent T-cell responses activated by Hepatitis B surface antigen mRNA vaccines. (**a**) BALB/3T3 cells were transiently transfected with pD2531.L, pD2531.M, and pD2531.S plasmids (1:1:1 mass ratio) via Lipofectamine 3000, and FCM was performed to detect pre-S2 antigen and S antigen expression on the cell surface. (**b**) After immunization of BALB/c mice with different hepatitis B surface antigen mRNA vaccines or PBS, splenic CD4^+^ T cells were isolated and stimulated with BALB/3T3 cells transiently expressing the LMS antigen. Meanwhile, antigen-incubated T cells from PBS-immunized mice were used as negative control and CD3/CD28 bead-stimulated T cells from PBS-immunized mice were used as positive control. FCM was used to measure the level of IFN-γ in CD4^+^ T cells after the addition of brefeldin A.

To further verify whether the hepatitis B surface antigen mRNA vaccines activated antigen-dependent T-cell responses, we immunized BALB/c mice with PBS, LMS mRNA, LS mRNA, or S mRNA. After 7 days, we isolated CD3^+^ T cells from immunized mice and stimulated the T cells in vitro with LMS-expressing BALB/3T3 cells constructed by transient transduction of plasmids. (Figure 5a). FCM analysis of CD3^+^ T cells intracellularly expressing IFN-γ is shown in Figure 5b and Figure S12. As with CD3/CD28 bead stimulated T cells, T cells in the mRNA vaccinated groups showed significant increase in IFN-γ expression following stimulation with the LMS antigen presented at the cell membrane. These findings indicate that the mRNA vaccines effectively activated the hepatitis B surface antigen-dependent T-cell response.

### 3.5. LMS mRNA Therapeutic Vaccines Exhibit Biosafety in Mice

According to our results, the LMS mRNA vaccine has the potential to break immune tolerance and to function to cure CHB. Therefore, acute toxicity tests were conducted in mice to further assess the safety of the vaccine. ICR mice were intramuscularly administered PBS or different doses of the LMS mRNA, which were approximately 5, 25, and 125 times the human dose of 100 μg of vaccine on a body-surface basis. Following two immunizations over a 21-day period, all the mice survived, and there were no significant changes in the viability or appearance of all mice at the end of the experiment. Following each injection, the mice treated with 7.5 μg or 37.5 μg exhibited swelling at the injection site. The swelling disappeared within one day, and the behavioral activity returned to normal. In addition, the body weight of the mice vaccinated with 37.5 μg of mRNA was significantly lower than that of the control mice on Day 3 (Figure S13a), and the condition returned to normal at subsequent time. A comparison of organ weights is presented in Figure S13b. A significant increase in spleen weight was observed in mice vaccinated with 37.5 μg of mRNA, which may be related to immune activation mediated by high antigen expression.

The ALT and AST results were not significantly different between the groups, indicating that the vaccine did not affect the liver function of the mice (Figure S14a). Renal function, including UREA, UA, and CR, was also normal (Figure S14b). Moreover, pathological analysis of the major organs revealed no significant toxicity (Figure S15). In conclusion, the acute toxicity test results indicate that the LMS mRNA vaccine does not cause significant toxicity in mice and predicts a favorable safety profile for clinical trials.

## 4. Discussion

The functional cure of CHB is not feasible without a breakthrough in immune tolerance, and some progress has been made through the use of immunomodulatory factors. For example, the activation of HBsAg-specific T cells by engineered anti-PDL1-IFNalpha heterodimers targeting the liver resulted in the disruption of immune tolerance in hepatitis B mice [30]. Mon et al. reported that immune checkpoint inhibitors (ICIs) could facilitate the clearance of serum HBsAg in hepatocellular carcinoma patients with HBsAg levels less than 100 IU/mL [31]. The substantial expression and secretion of HBsAg particles play pivotal roles in the onset of humoral immune tolerance [6,8,32], and it has been proposed that low levels of HBsAg may create a therapeutic window for therapeutic vaccines or drugs for hepatitis B [33]. This finding indicates that baseline levels of HBsAg impact the efficacy of drugs in breaking immune tolerance, as further evidenced by data from the immunization experiment of rAAV8 HBV1.3 mice in our study. Therefore, it is important to control serum HBsAg first to overcome immune tolerance in CHB patients.

Direct use of anti-HBsAg antibodies has been demonstrated to control HBsAg in mice [34], which could control serum HBsAg in the pretreatment phase of CHB. While the recombinant HBsAg protein is efficient when used as a prophylactic vaccine, it is not a viable therapeutic vaccine for curing hepatitis B [35]. Vandepapeliere et al. demonstrated that the combination of lamivudine and the HBsAg/AS02 vaccine resulted in a significantly higher rate of HBe seroconversion in CHB patients than lamivudine alone [36]. Furthermore, Backes et al. discovered that a protein-prime/MVA-boost vaccination regimen could effectively overcome HBV-specific tolerance in HBV Tg mice with low or medium levels of HBsAg [37]. Additionally, optimization of the adjuvant formulation and utilization of a heterologous HBsAg subtype could induce HBV-specific T cells and antibodies in HBV Tg mice with high HBsAg levels [37]. These studies demonstrate that the efficacy of anti-HBsAg antibodies or HBsAg vaccines in the treatment of CHB is significantly influenced by the serum HBsAg level, selection of adjuvants, and methods of immunization. In contrast, we designed therapeutic mRNA vaccines that is capable of expressing and presenting the structural pre-S antigen in vivo, which could effectively block HBV infection and alleviate the exhaustion of HBsAb and B cells. The process of HBsAb production was not influenced by high levels of HBsAg.

Research on therapeutic vaccines for hepatitis B based on pre-S has been conducted for many years. For example, the administration of the preS1 peptide to HBV Tg mice resulted in the clearance of HBV and a reduction in HBsAg-mediated immune tolerance by inducing anti-preS1 antibodies [38]. Sequential administration of the preS1 peptide and HBsAg vaccine induced HBsAg/HBsAb seroconversion in some mice [38], but it did not exhibit therapeutic effects in the clinic. In this study, anti-pre-S antibodies were detected in the serum of HBV Tg mice immunized with the pre-S peptide, but the IgG was unable to recognize LMS VLP containing the pre-S antigen and did not result in a breakthrough of immune tolerance in HBV Tg mice. Combined with previous studies, our findings suggest that this result is attributable to the absence of a spatial structure for the pre-S peptide, which impairs the ability of the peptide to induce effective antibodies. Zhu et al. demonstrated that the fusion of IgV_CTLA-4 with L protein did not disrupt the formation of T and B-cell epitopes of the L protein and that it exhibits good immunogenicity [39]. Accordingly, the S protein has been shown to facilitate L protein expression and secretion via VLPs or at the cell membrane by interacting with each other [20], which was further confirmed by our results, and we found that the presence of M protein could contribute to the stability of pre-S-containing VLPs. Additionally, Yu et al. reported that neuropilin-1 (NRP1), a novel host factor that mediates HBV infection, regulates HBV entry into hepatocytes by interacting with preS1 and NTCP [40], which may be a new target used to block HBV infection.

LMS VLP combined with the L-pampo adjuvant were reported to elicit a robust antibody response in three types of HBV Tg mice, with clearance of the HBV antigen [29]. In contrast, although we also detected high HBsAb responses in BALB/c HBV Tg mice, HBsAg was not cleared, and a reduction in serum HBsAg was observed only in some mice with relatively low antigen levels (<10,000 IU/mL). This result may be related to the fact that the immunity activation effect of aluminum adjuvant is significantly less than that of L-pampo, and the level of HBsAg in selected HBV Tg mice appears to be considerably greater than that reported previously. In addition, we also found that designed mRNA therapeutic vaccines were able to activate high levels of HBsAb faster or earlier, possibly because of the stronger adjuvant effect and more effective antigen presentation. Studies have shown that the third-generation pre-S/S vaccine (Sci-B-Vac/PreHevbrio) achieves serologic conversion only in low-level HBsAg carriers (<500 lU/mL) [33]. Pham et al. reported that the S/pre-S1/pre-S2 vaccine in combination with lamivudine was significantly more effective than lamivudine alone in the short term [22]. Similarly, anti-pre-S1/pre-S2 antibodies as well as antigen-specific IFN-γ+ T cells were observed only with combination treatment with the S/pre-S1/pre-S2 vaccine and IFN-α [41]. In this study, the LMS mRNA vaccine was observed to achieve both HBsAg seroconversion and HBV DNA clearance without additional agents or adjuvants. Furthermore, in vitro studies demonstrated that the L, M, and S proteins were predominantly expressed in the cell membrane and that VLPs were undetected in the supernatant. Concurrently, we found that if the screened monoclonal cells had a weak ability to secrete VLPs, the expression of the pre-S antigen on the cell surface increased.

In addition to VLPs, numerous studies have designed HBV therapeutic vaccines using alternative vectors [42]. For example, the presentation of preS1 to SIGNR1(+) DC cells and lymphoid-associated SIGNR1(+) cells via ferritin nanoparticles induced high levels of anti-preS1 antibodies and achieved viral clearance in HBV mice [10]. Similarly, a peptide vaccine designed on the basis of nanoliposomes enhanced HBeAg seroconversion in CHB patients [43]. Furthermore, lentiviral vectors (LVs) encoding the HBV core, preS1, or large HBsAg (LHB) proteins induced a decrease in serum HBsAg levels in 60% of hepatitis B mice [44]. In recent years, studies on the treatment of hepatitis B or hepatocellular carcinoma with the help of mRNA technology, including the delivery of IL2 cytokines and engineered TCR-T cells, have rapidly emerged [45,46].

Currently, there are few studies on therapeutic mRNA vaccines for CHB. Zhao et al. achieved seroconversion in a hepatitis B mouse model via an mRNA vaccine encoding full-length HBsAg and analyzed the activation of the innate immune response, the HBsAg-specific T-cell immune response, and the memory B-cell response [47]. We designed a multivalent mRNA therapeutic vaccine based on three hepatitis B surface antigens, which also contain S mRNA. However, despite the superiority of our vaccine in terms of antibody production time and level, the final HBsAg clearance was not advantageous, which may be due to the better immune response of T cells activated by S mRNA. More importantly, our vaccine markedly stimulated antibody responses against pre-S antigens. Pooter et al. developed a multivalent mRNA vaccine encoding the HBV core, polymerase, and preS2-S antigens, which elicited a robust T-cell response and partially reduced serum HBsAg levels in hepatitis B mice [48]. Based on these results in two AAV HBV mouse models, we propose that for CHB patients with high levels of HBsAg, it is important to not only activate HBsAb to break immune tolerance but also the T-cell immune response to further clear HBV. In this study, we employed a similar approach to immunize mice with co-formulated mRNAs. In a parallel study, Pooter et al. conducted a comparative analysis of immune responses between co-formulated mRNAs and co-encapsulated mRNAs [48]. The latter consist of different mRNAs encapsulated simultaneously in a single batch of LNP. Although their results showed no significant difference in immune activation between two forms of mRNA vaccines, it has also been shown that the efficiency of co-transfection of multiple mRNAs in different ways may lead to expression heterogeneity [49]. In addition, different LNP formulations have effects on organ/cell targeting, protein expression efficiency, and immune activation, and the physicochemical properties of the particles, such as particle size and ZP, also determine the delivery efficiency. Therefore, further refinement and in-depth studies are imperative to address the aforementioned issues. Finally, the activation of different types of immune responses in the prime and boost phases of vaccination may exert better therapeutic effects [37].

Elevated levels of HBsAg can induce antigen-mediated immune tolerance, resulting in impaired plasma cell differentiation [6,50]. Qi et al. reported that in the early stage of immunization, antigen-specific B cells can establish germinal centers (GCs) and differentiate into plasma cells and memory B cells in mice, but antibodies then decline significantly [50]. A study on PEG-IFNα demonstrated differential alterations in antigen-specific B-cell phenotypes and subtypes, and CD40L+ Th cells were associated with the positive recovery of HBsAg-specific B cells [51]. Bailey et al. reported that CD40L activates the antiviral immune response of CD8^+^ T cells, which is influenced by CD4^+^ T cells [52]. Therefore, we concluded that the antibody response can be further enhanced by the activation of supportive signaling in B cells and that the inhibitory effect of CD4^+^ Th cells is needed. The T-cell immune response has been less studied in this research, and we detected antigen-dependent T-cell activation in mice immunized with mRNA vaccines. Recently, Andreata et al. reported that antiviral activity was enhanced by 4-1BB stimulation [53]. Bosch et al. reported that CD8^+^ T cells associated with hepatic sinusoidal endothelial cells by recognizing viral antigens expressed on hepatocytes, which enhanced T-cell cAMP–PKA signaling and led to T-cell dysfunction [54].

Finally, given that HBV does not infect mouse hepatocytes, we employed hepatitis B replication mouse models, rather than infection models, which are unable to effectively represent the process of HBV binding to NTCP receptors and subsequent infection of hepatocytes. Therefore, our study was unable to fully validate the mechanism by which the vaccine breaks immune tolerance by blocking the binding of pre-S to NTCP in vivo. To address this limitation, the in vitro HBV infection inhibition assay is needed to indirectly verify whether the therapeutic vaccine induced a specific antibody response against HBV infection.

## 5. Conclusions

**Figure 6:**
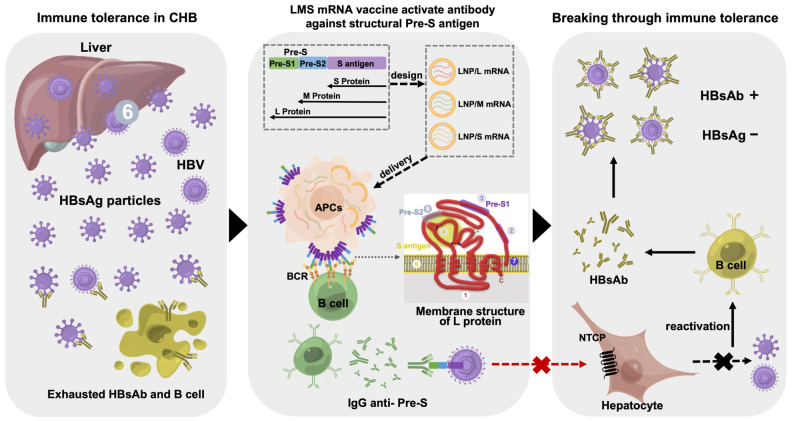
Schematic diagram of the mechanism by which the LMS mRNA therapeutic vaccine disrupts HBsAg-mediated immune tolerance and reactivates immune responses against HBV. The numbers 1–6 marked on the membrane structure of L protein represent intracellular structure of S antigen, T cell recognition epitopes of pre-S1 antigen, hepatocyte surface receptor NTCP binding site for pre-S1 antigen, S antigen (highly variable region) with a-antigen determinant, lipid membrane, hepatocyte binding region for pre-S2 antigen, membrane attachment region of HBV-infected hepatocytes, respectively.

In summary, a trivalent hepatitis B therapeutic vaccine, LMS mRNA, was designed to present structural pre-S antigens on the cell membrane in support of the S protein and M protein. In HBV Tg mice with high levels of HBsAg, LMS mRNA activated the production of high levels of HBsAb, disrupted immune tolerance and stimulated the production of high-affinity IgG antibodies against structural pre-S. Moreover, the vaccine markedly reduced or eliminated serum HBsAg and HBV DNA in two hepatitis B mouse models with the production of HBsAb. Furthermore, the vaccine effectively activated antigen-specific antibody responses and T-cell responses. A schematic diagram of the mechanism by which LMS mRNA vaccines cure CHB is presented in Figure 6. Additionally, the vaccine was verified to be safe. Consequently, LMS mRNA can be employed as a novel therapeutic vaccine for CHB, and it has the potential to achieve a functional cure for hepatitis B.

## Data Availability

The datasets generated during and/or analyzed during the current study are available from the corresponding author on reasonable request.

## Supplementary Materials

The following supporting information can be downloaded at: https://www.mdpi.com/article/10.3390/pharmaceutics17020211/s1, Sequence data: Schematic representation of hepatitis B surface antigen proteins and membrane structure of the L-protein; Table S1: Characterization of LNP/mRNA encoding hepatitis B surface antigens; Figure S1: Schematic representation of hepatitis B surface antigen proteins and membrane structure of the L-protein; Figure S2: Analysis of different hepatitis B surface antigen proteins in LMS VLP and TEM characterization; Figure S3: Identification of HEK293F monoclonal cells expressing secreted VLPs containing L and S proteins; Figure S4: Analysis of L protein expression on the cell surface after transduction of hepatitis B surface antigens; Figure S5: Identification of HEK293F monoclonal cells expressing the L protein at the cell membrane; Figure S6: Analysis of the L protein and membrane raft structure in different 293F monoclonal cells; Figure S7: Purification and analysis of pre-S protein; Figure S8: Analysis of changes in serum HBsAb for HBV Tg mice immunized with different combinations of mRNAs encoding hepatitis B surface antigens; Figure S9: Analysis of changes in serum HBsAg for HBV Tg mice immunized with different combinations of mRNAs encoding hepatitis B surface antigens; Figure S10: Analysis of changes in serum HBsAg in pAAV HBV1.2 mice immunized with different hepatitis B surface antigen mRNA vaccines or GFP mRNA at week 0 and week 9; Figure S11: Serological response analysis of rAAV8 HBV1.3 mice immunized with LMS mRNA vaccines; Figure S12: Representative gating of splenic CD4^+^ T cells intracellularly expressing IFN-γ; Figure S13: Acute toxicity test of LMS mRNA vaccines in mice; Figure S14: Liver and renal function analysis in acute toxicity tests of LMS mRNA vaccines; Figure S15: H&E staining and pathologic analysis of major organs (heart, liver, spleen, lungs, and kidney) in mice vaccinated with different doses of LMS mRNA vaccines or PBS at the end of the acute toxicity test.
