# Supplementary material for: A Multivalent mRNA Therapeutic Vaccine Exhibits Breakthroughs in Immune Tolerance and Virological Suppression of HBV by Stably Presenting the Pre-S Antigen on the Cell Membrane
Source: Pharmaceutics. 2025 Feb 7;17(2):211. doi: 10.3390/pharmaceutics17020211 (PMC11859219; doi:10.3390/pharmaceutics17020211)
Supplement: Supplementary file 1 [file pharmaceutics-17-00211-s001.zip › pharmaceutics-3397699-supplementary.pdf]

pharmaceutics

Supplementary Material: A Multivalent mRNA Therapeutic Vaccine Exhibits Breakthroughs in Immune Tolerance and Virological Suppression of HBV by Stably Presenting the Pre-S Antigen on the Cell Membrane

Shang Liu, Jie Wang, Yunxuan Li, Muhan Wang, Pei Du, Zhijie Zhang, Wenguo Li, Rongchen Sun, Mingtao Fan, Meijia Yang and Hongping Yin

Sequence data

Primary amino acid sequence of HBV surface antigen large protein (L protein)

MGGWSSKPRKGMGTNLSVPNPLGFFPDHQLDPAFKANSENPDWDLNPHKDN
WPDANKVGVGAFGPGFTPPHGGLLGWSPQAQGLLTTVPAAPPPASTNRQSGRQPTP
LSPPLRDTHPQAMQWNSTTFHQTLQDPRVRALYFPAGGSSSGTVSPAQNTVSAISSILS
KTGDPVPNMENIASGLLGPLLVLQAGFFLLTKILTIPQSLDSWWTSLNFLGGTPVCLGQ
NSQSQISSHSPTCCPPICPGYRWMCLRRFIIFLCILLLCLIFLLVLLDYQGMLPVCPLIPGS
STTSTGPCKTCTTPAQGTSMFPSCCCTKPTDGNCTCIPIPSSWAFAKYLWEWASVRFSW
LSLLVPFVQWFVGLSPTVWLSVIWMMWFWGPSLYNILSPFMPLLPIFFCLWVYI.

The pre-S1 sequence is shown in black, the pre-S2 sequence is shown in green, and the S antigen sequence is shown in yellow. The L protein contains full-length sequence. HBV surface antigen middle protein (M protein) contains pre-S2 sequence and S antigen sequence. HBV surface antigen small protein (S protein) contains only the S antigen sequence.

Table S1. Characterization of LNP/mRNA encoding hepatitis B surface antigens.

	LNP/L mRNA	LNP/M mRNA	LNP/S mRNA
Appearance	Clear & Transparent or Ivory Suspension	Clear & Transparent or Ivory Suspension	Clear & Transparent or Ivory Suspension
Encapsulation Efficiency	96.12%	92.34%	95.65%
mRNA Concentration	262.37 μg/mL	342.95 μg/mL	248.26 μg/mL
Average particle Size	79.59 nm	82.95 nm	84.58 nm
Polydispersity Index (PDI)	0.11	0.04	0.08
pH	7.03	7.28	7.05
Zeta Potential	−5.30 mV	−7.93 mV	−3.28 mV
Endotoxin	≤10 EU/mL	≤10 EU/mL	≤10 EU/mL

 https://doi.org/10.3390/pharmaceutics17020211

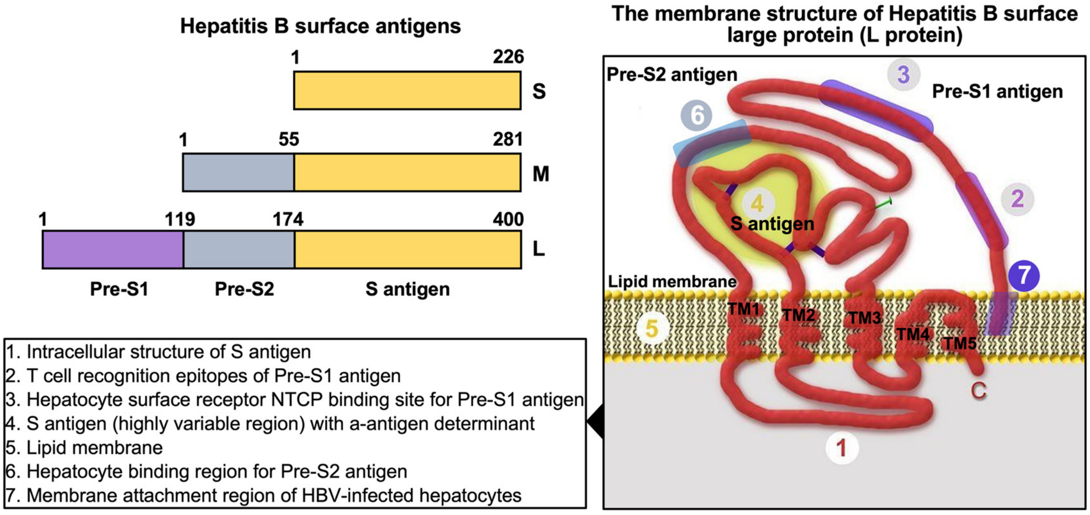

Figure S1. Schematic representation of hepatitis B surface antigen proteins and membrane structure of the L-protein. Hepatitis B surface antigens are composed of L protein, M protein, and S protein, which are encoded by the same variable reading frame. The M protein contains a pre-S2 domain in the N-terminus and an S protein region in the C-terminus, and the extension of the N-terminus with the pre-S1 domain results in the formation of the L protein. Functionally, the S antigen provides five transmembrane (TM) regions and contains highly variable region. In addition, the S antigen can bind to acetyl heparin sulfate on the surface of hepatocytes via an a-antigen determinant cluster. pre-S1 and pre-S2 antigens mediate the binding of HBV to the hepatocyte surface receptor sodium taurocholate cotransporter polypeptide (NTCP). The T cell recognition antigenic epitope in pre-S1 and the membrane attachment region of HBV-infected hepatocytes are also shown in figure on the right panel.

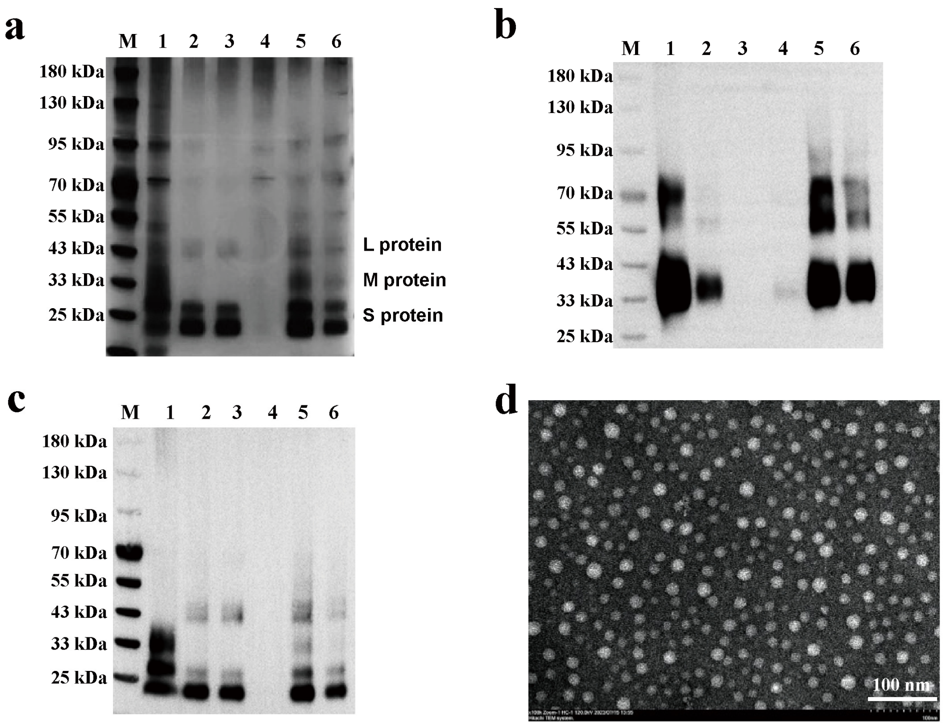

Figure S2. Analysis of different hepatitis B surface antigen proteins in LMS VLP and TEM characterization. After fermentation of LMS-A4 293F cells, the supernatant was collected, pretreated, and then purified via SEC and virus purification columns. Samples from the supernatant concentrate (line 1), first peak of the SEC (line 2), second peak of the SEC (line 3), third peak of the SEC (line 4), viral purification column collection I (line 5), and viral purification column collection II (line 6) were subjected to SDS–PAGE. (**a**) Silver-staining was performed to analyze the purity of

the VLP proteins, including the L protein (38 kDa, 42 kDa), M protein (33 kDa, 36 kDa) and S protein (24, 27 kDa). (**b**) WB (anti-pre-S) and (**c**) WB (anti-S antigen), to analyze protein specificity. (**d**) TEM detection of purified and concentrated VLPs.

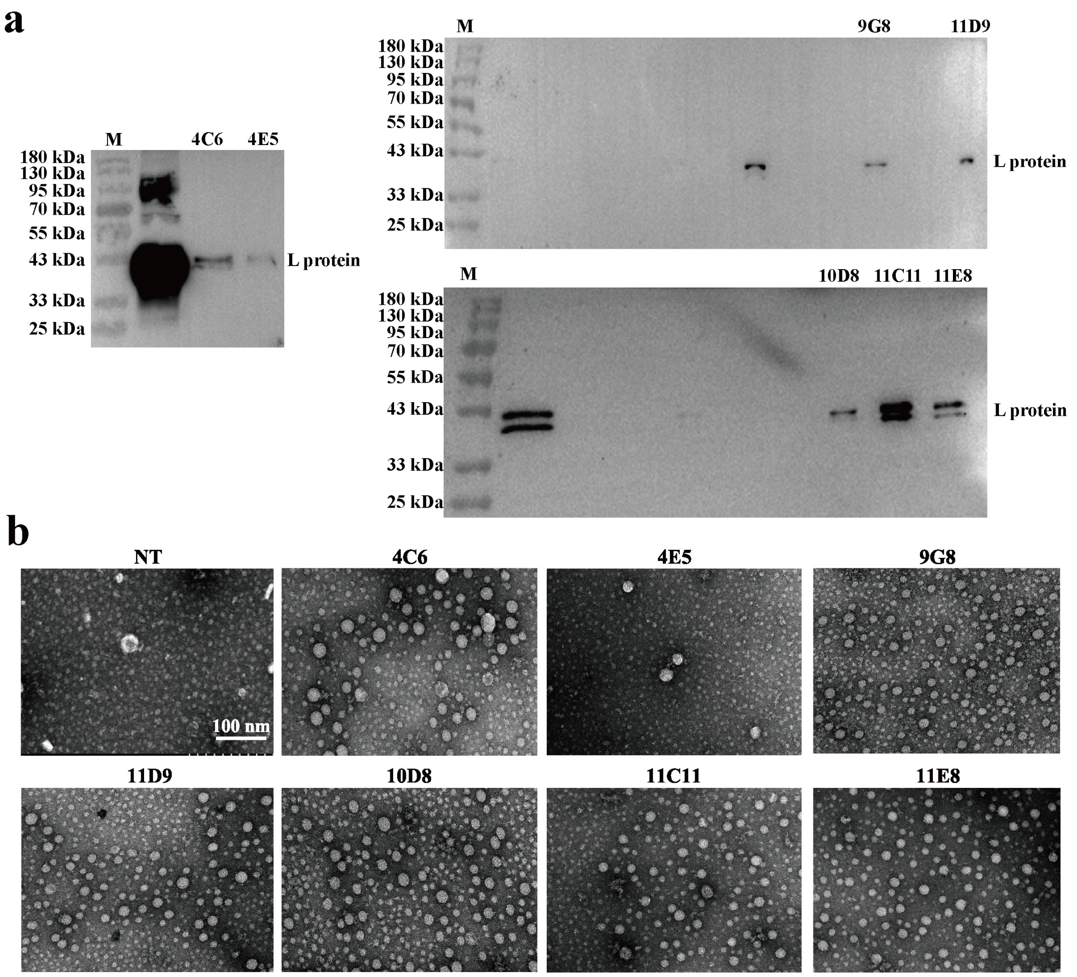

Figure S3. Identification of HEK293F monoclonal cells expressing secreted VLPs containing L and S proteins. The pD2531.L and pD2531.S plasmids were stably transfected into GS-HEK293F cells, which were subjected to Gln-deficient pressurization screening. Monoclonal cells were selected, and culture supernatants were collected for (**a**) WB (anti-pre-S2 antigen) detection of the L protein and (**b**) TEM detection of VLPs in the supernatants.

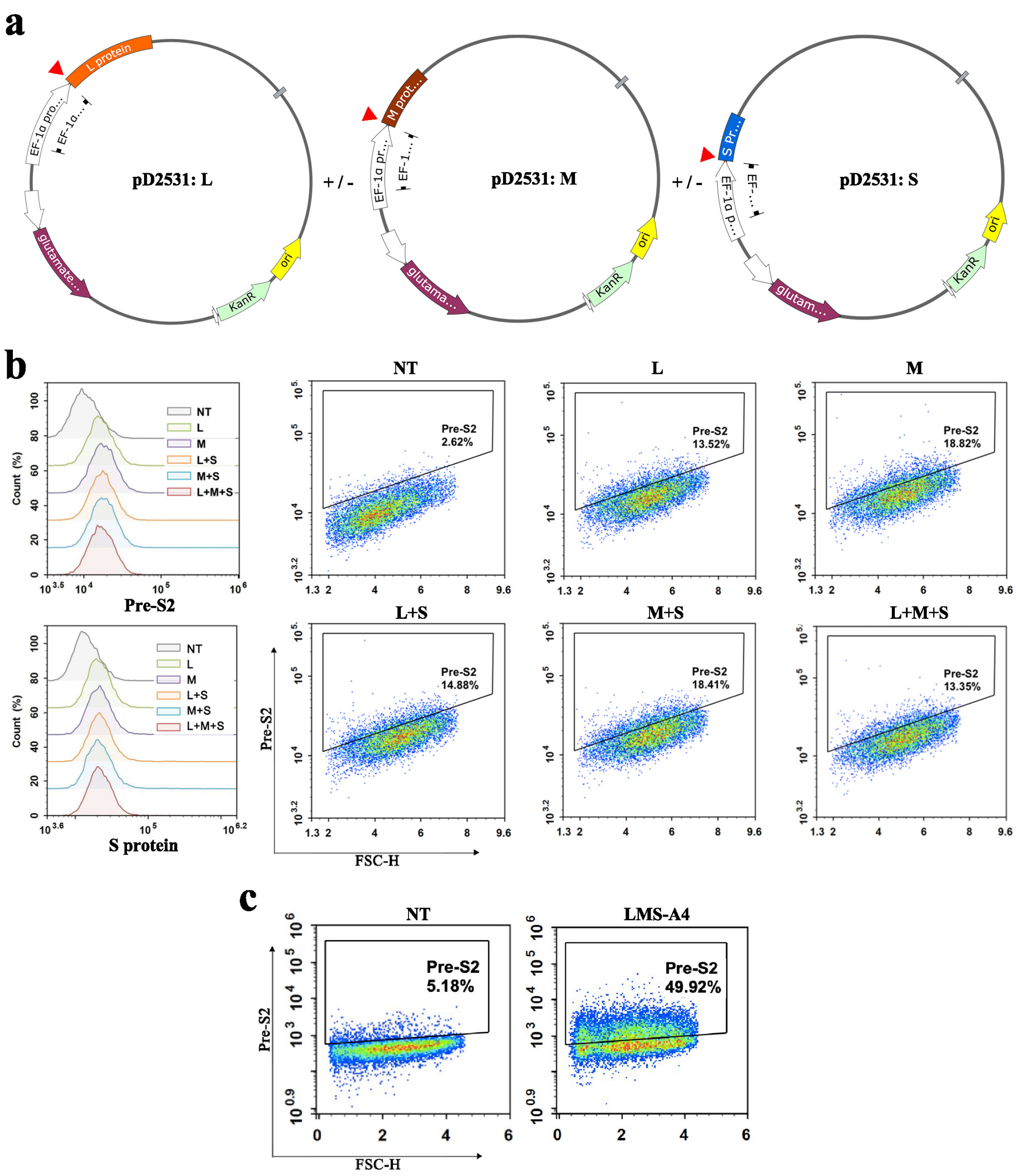

Figure S4. Analysis of L protein expression on the cell surface after transduction of hepatitis B surface antigens. (**a**) Schematic representation of three constructed hepatitis B surface antigen expression plasmids, pD2531.L, pD2531.M and pD2531.S. The three plasmids were transfected into 293T cells in different combinations (mass ratio of 1:1 or 1:1:1) via Lipofectamine 3000. (**b**) FCM analysis of the expression of pre-S2 and S antigens on the surface of cells after 48 h of transfection. (**c**) Percentage of LMS-A4 293F cells expressing pre-S2 antigen on the surface, as detected by FCM.

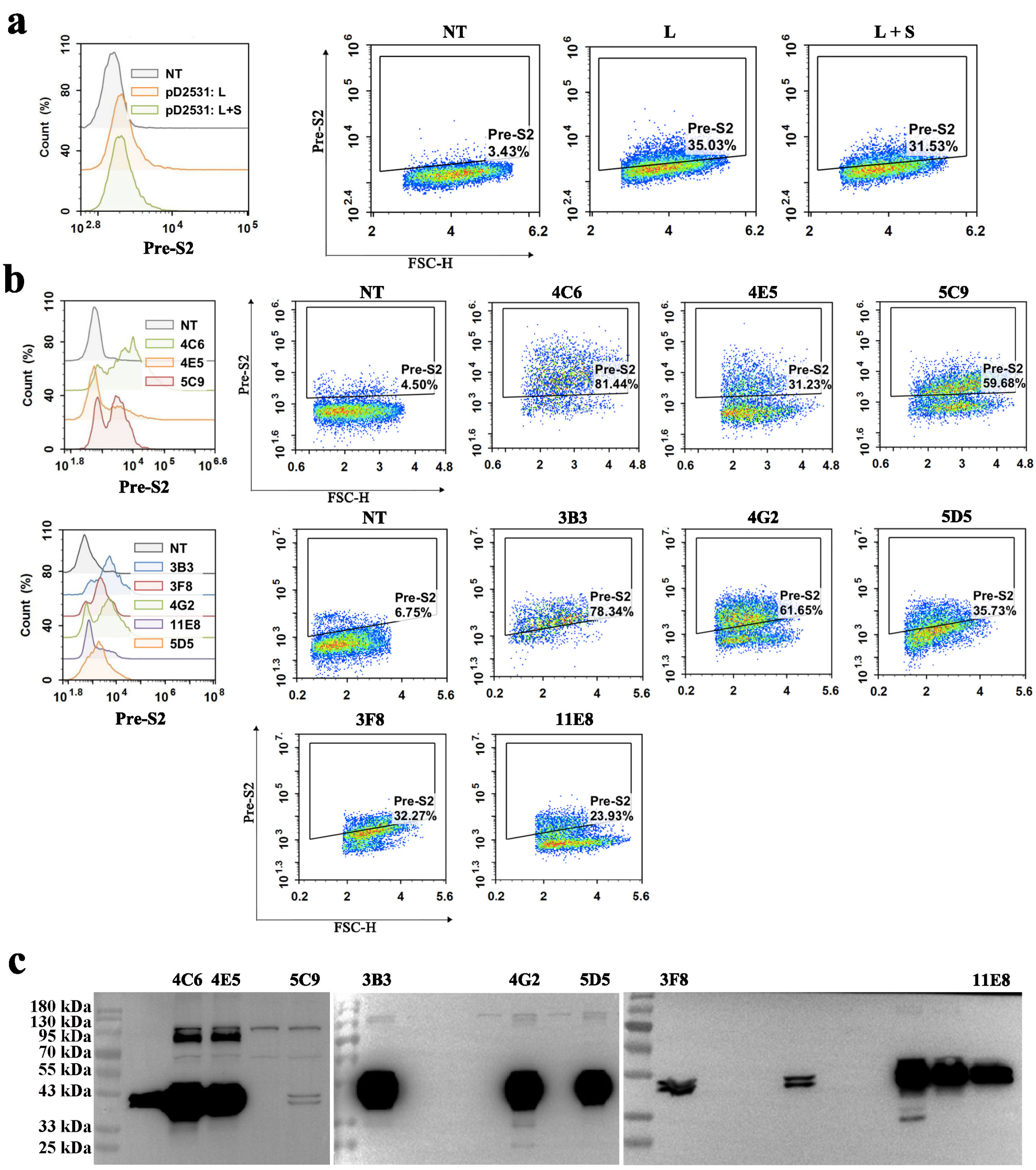

Figure S5. Identification of HEK293F monoclonal cells expressing the L protein at the cell membrane. The pD2531.L and pD253.S plasmids were stably transfected into Gln-HEK293F cells, which were subjected to a Gln-deficient pressurization screen. (**a**) FCM was used to detect the expression of the pre-S2 antigen on the surface of pooled cells. (**b**) Monoclonal cells were selected, and FCM was performed to detect the expression of the pre-S2 antigen on the surface of monoclonal cells. (**c**) WB (anti-pre-S2 antigen) was performed to detect the expression of the L protein at membrane of monoclonal cells.

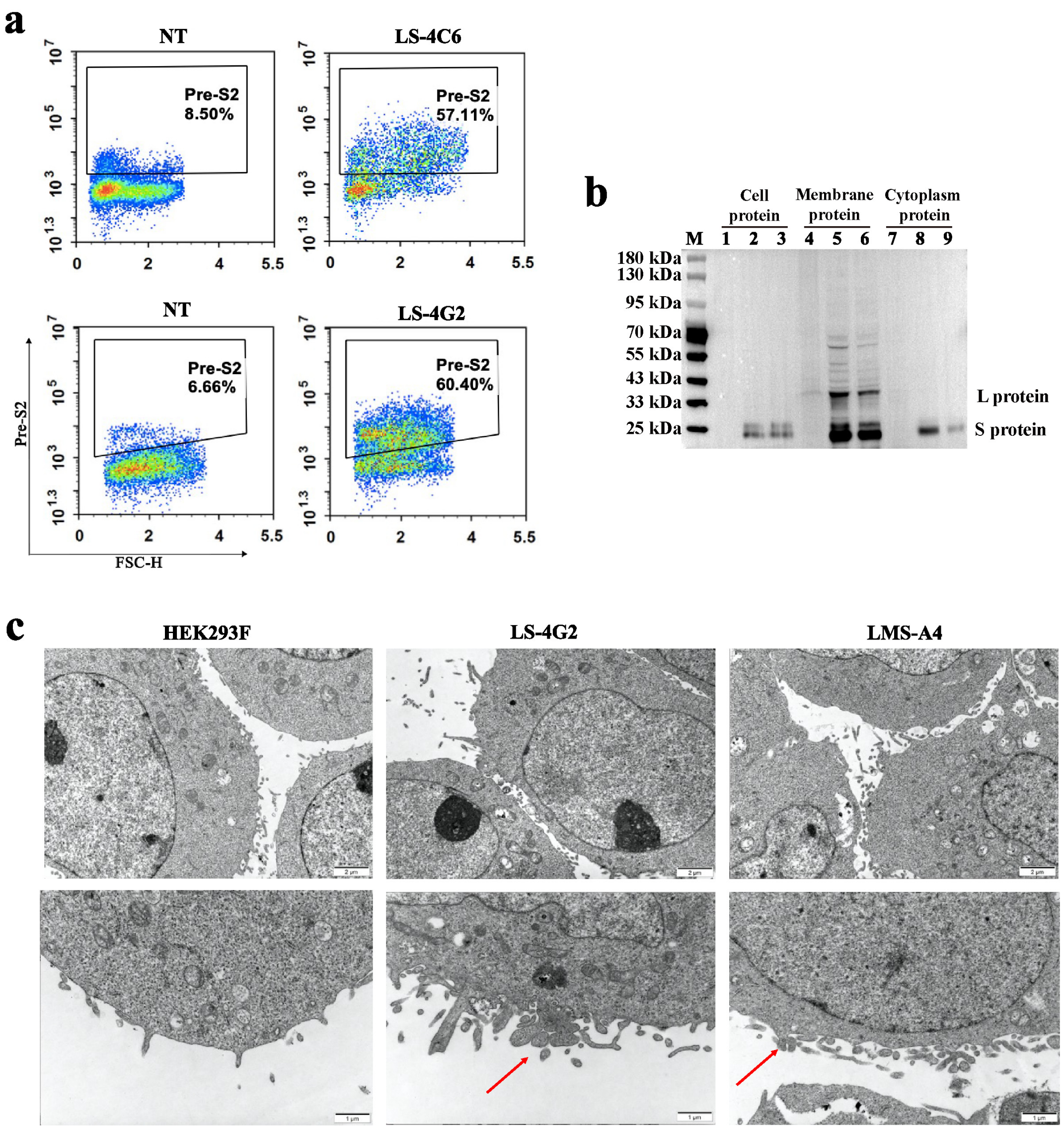

Figure S6. Analysis of the L protein and membrane raft structure in different 293F monoclonal cells. (**a**) Proportion of cells expressing pre-S2 antigen detected by FCM after multiple passaging cultures of LS-4C6 293F and LS-4G2 293F cells. (**b**) WB (anti-S antigen) was used to detect the expression of L and S proteins in different cell components of HEK293F (lines 1, 4 and 7), LS-4C6 293F (lines 2, 5 and 8) and LS-4G2 293F (lines 3, 6 and 9) cells. (**c**) TEM image of membrane raft structures (indicated by red arrows) on the surface of HEK293F, LS-4C6 293F and LMS-A4 293F cells, below are partially enlarged images.

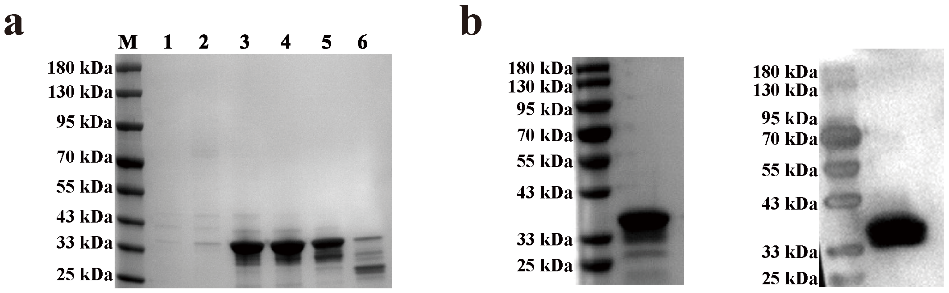

Figure S7. Purification and analysis of pre-S protein. (**a**) The *E. coli*-expressed pre-S peptide was purified via Ni column and SEC, and samples with different absorption peak positions were collected for SDS–PAGE and Coomassie brilliant blue staining. Lines 3-5 are the samples enriched

with the pre-S peptide. (**b**) Samples from lines 3-5 in panel a were mixed to remove endotoxin and subjected to SDS–PAGE with Coomassie brilliant blue staining (panel left) and WB (panel right) to detect the purity and specificity of the pre-S peptide, respectively.

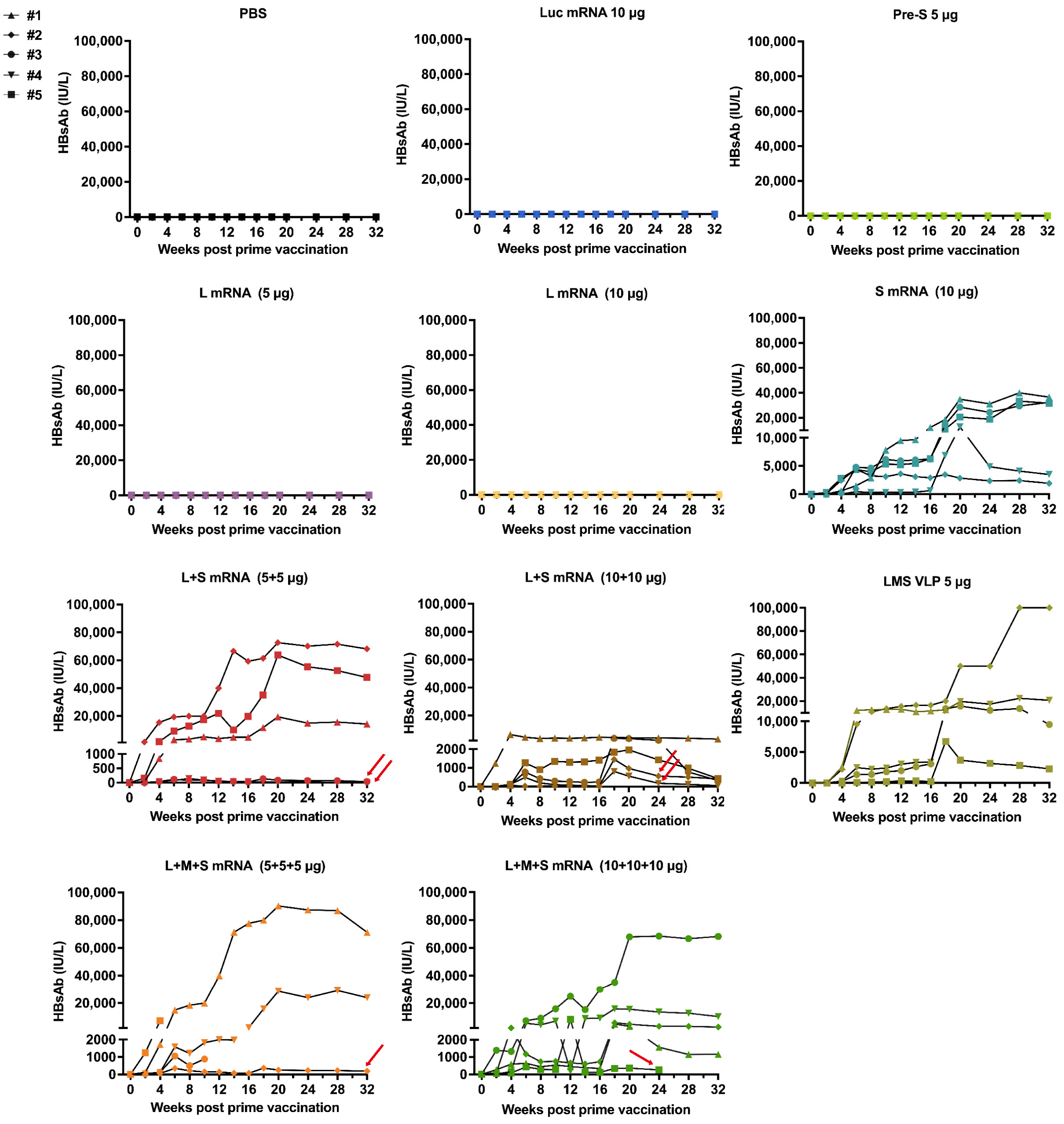

Figure S8. Analysis of changes in serum HBsAb for HBV Tg mice immunized with different combinations of mRNAs encoding hepatitis B surface antigens or with the control vaccine. Red arrows indicate changes of HBsAb levels in mice whose HBsAb levels were consistently less than 2,000 IU/L and greater than 0 IU/L.

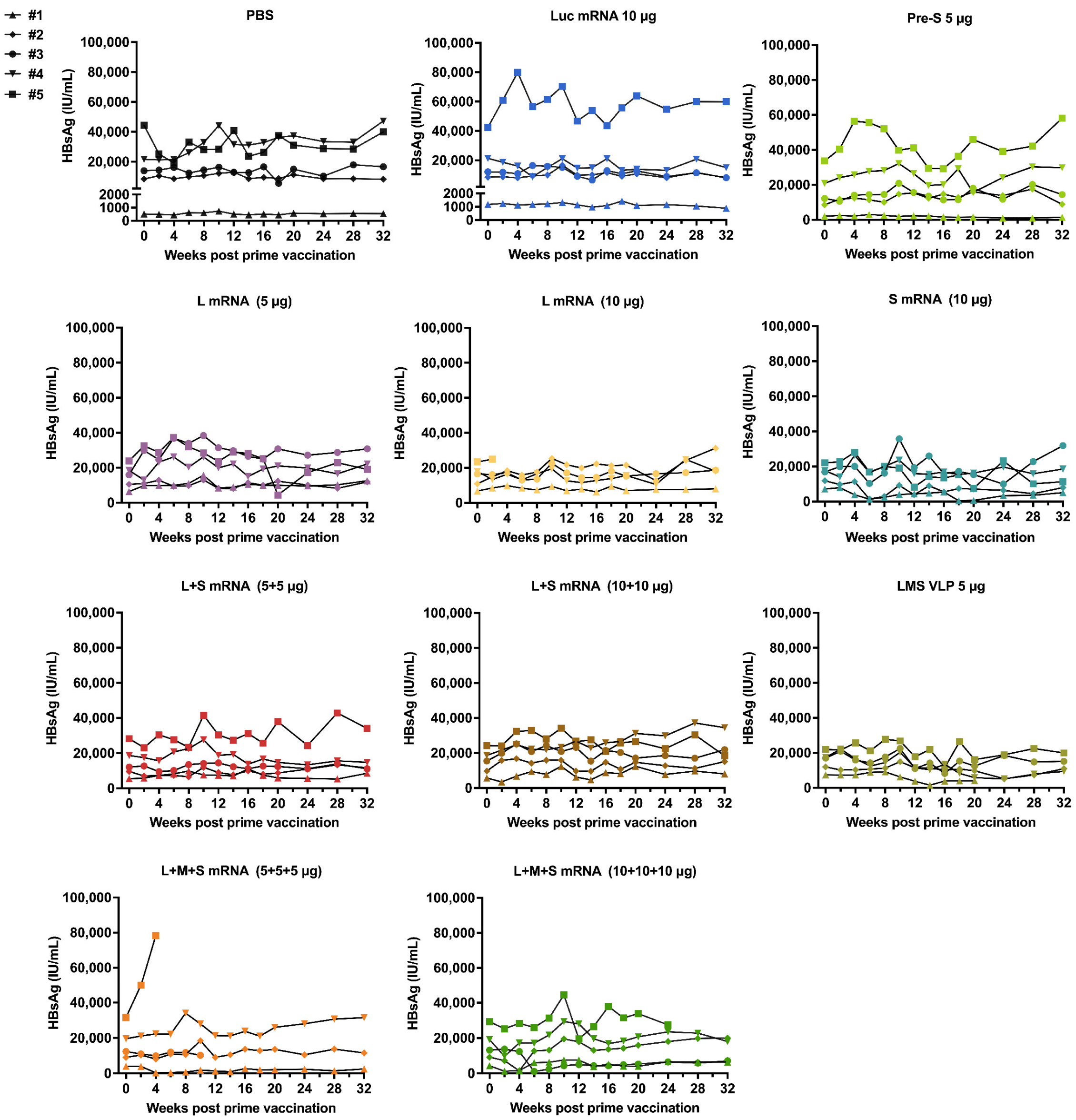

Figure S9. Analysis of changes in serum HBsAg for HBV Tg mice immunized with different combinations of mRNAs encoding hepatitis B surface antigens or with control vaccines.

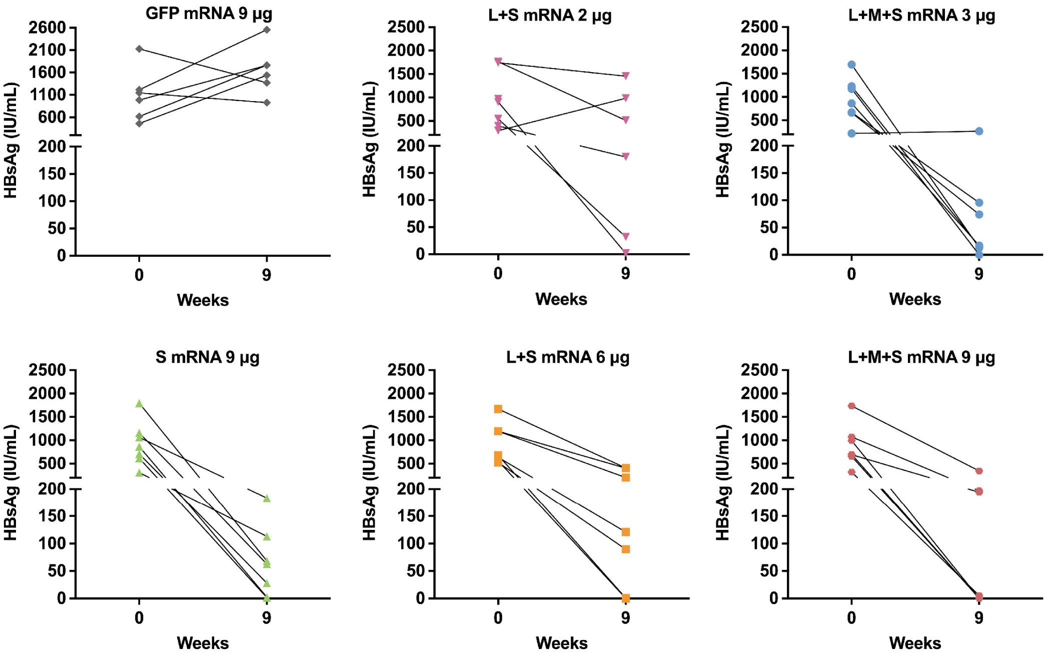

Figure S10. Analysis of changes in serum HBsAg in rAAV HBV1.3 mice immunized with different hepatitis B surface antigen mRNA vaccines or GFP mRNA at week 0 and week 9.

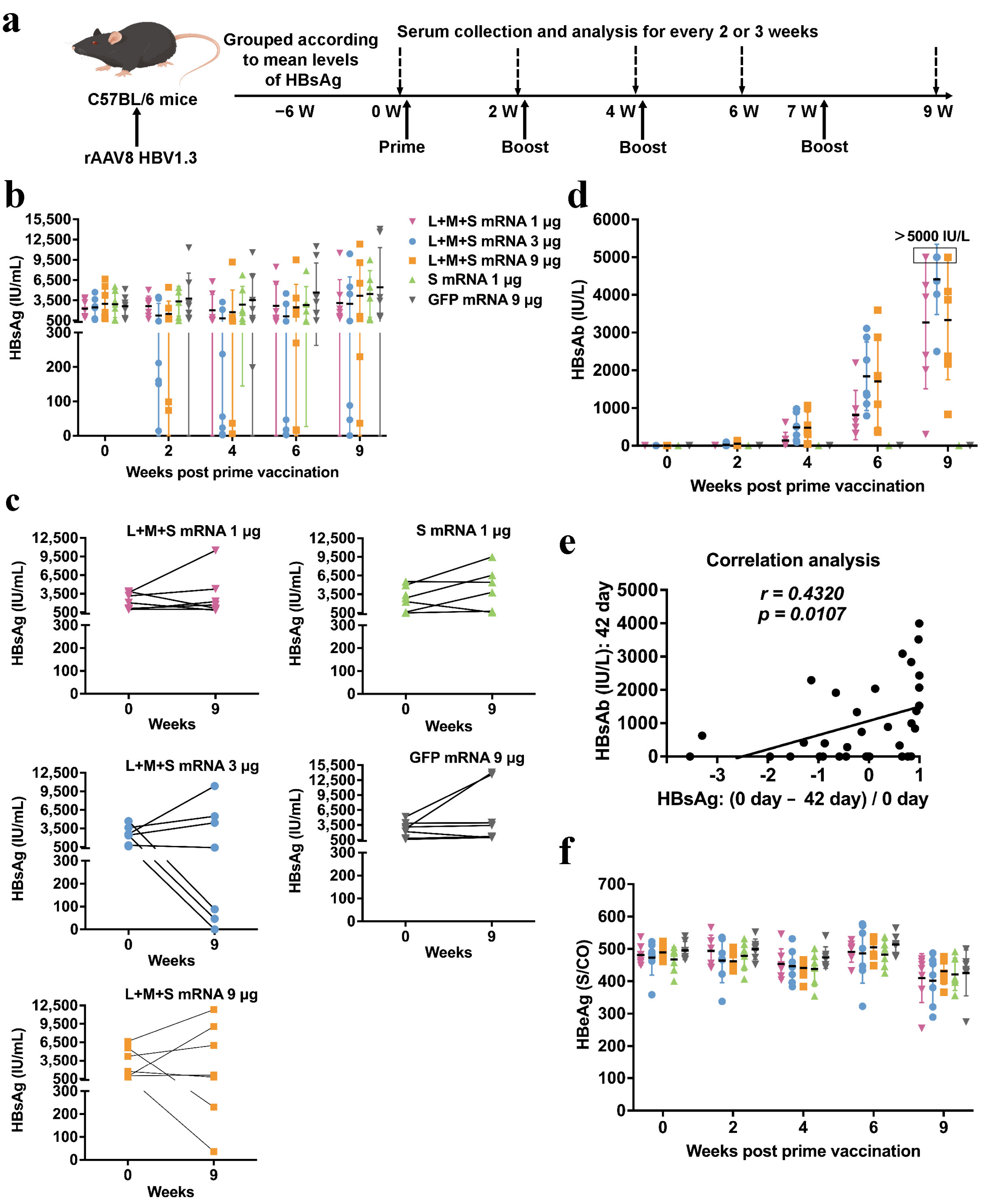

Figure S11. Serological response analysis of rAAV8 HBV1.3 mice immunized with LMS mRNA vaccines. (**a**) Schematic representation of the experimental protocol for immunization of rAAV8 HBV1.3 (5×10^{10} vg/mouse) mice with different doses of LMS mRNA, S mRNA, or GFP mRNA. Changes in serum (**b**) and (**c**) HBsAg and (**d**) HBsAb levels after 9 weeks of immunization in rAAV8 HBV1.3 mice. (**e**) Correlation analysis of the serum activation of HBsAb with reduced levels of HBsAg at week 0 and week 6. (**f**) Detection of serum HBeAg changes in the mice at weeks 0-9. Representative results are presented as the means ± standard deviations (SDs).

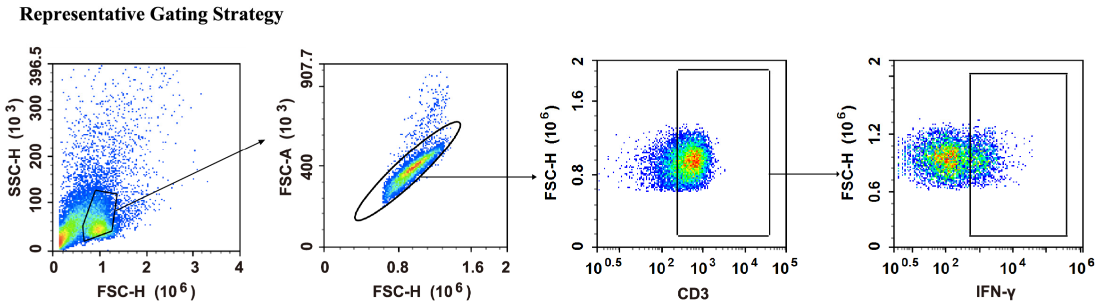

Figure S12. Representative gating of splenic CD4+ T cells intracellularly expressing IFN-γ stimulated by CD3/CD28 beads in the antigen-dependent T cell activation experiment. The number of cells in the gate in the left panel (FSC-H with SSC-H) is 10,000.

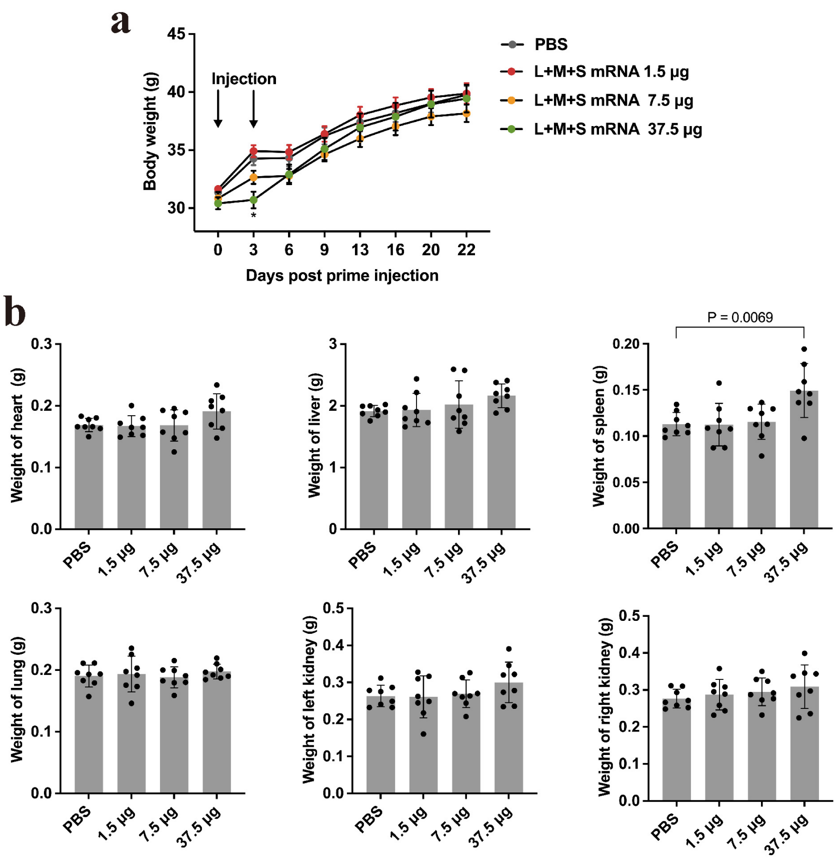

Figure S13. Acute toxicity test of LMS mRNA vaccines in mice. (**a**) Changes in the body weights of mice immunized with different doses of LMS mRNA or PBS. (**b**) Comparison of the weights of the heart, liver, spleen, lung, and kidney in the mice at the end of the experiment. The dots in figures represent the value of the corresponding indicator for each mice. Representative results are presented as the means ± standard deviations (SDs).

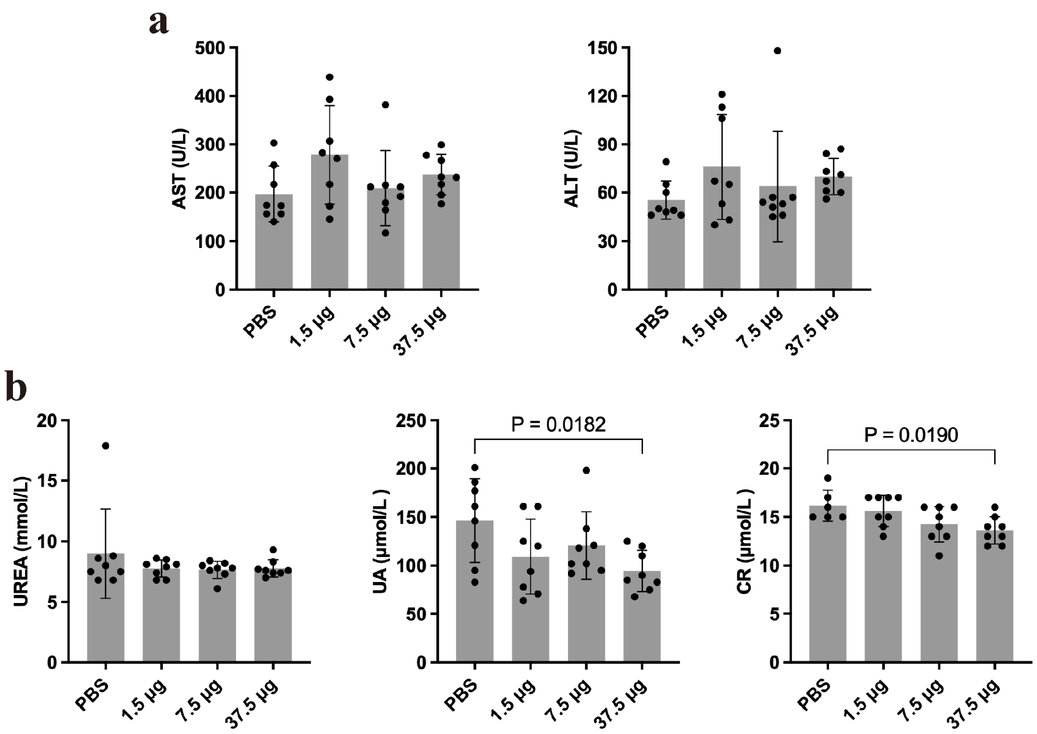

Figure S14. Liver and renal function analysis in acute toxicity tests of LMS mRNA vaccines. (**a**) Liver function indices, including aspartate aminotransferase (AST) and alanine aminotransferase (ALT) levels, were analyzed at the end of the acute toxicity test. (**b**) Renal function indices, including urea, uric acid (UA) and creatinine (CR) levels, were analyzed at the end of the acute toxicity test. The dots in figures represent the value of the corresponding indicator for each mice. Representative results are presented as the means ± standard deviations (SDs).

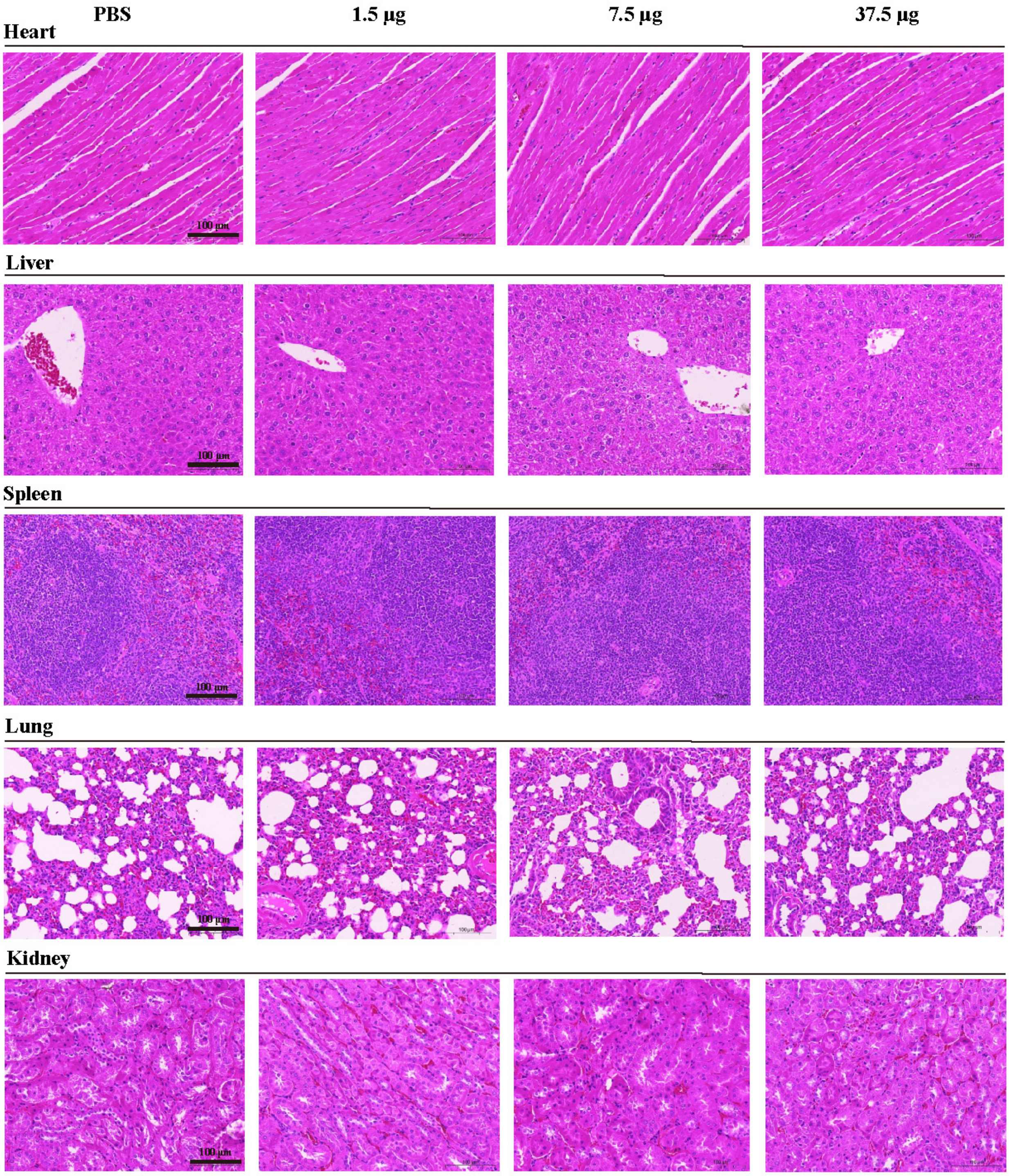

Figure S15. H&E staining and pathologic analysis of major organs (heart, liver, spleen, lungs, and kidney) in mice vaccinated with different doses of LMS mRNA or PBS at the end of the acute toxicity test.
